# Supplementary material for: USP22 regulates lipidome accumulation by stabilizing PPARγ in hepatocellular carcinoma
Source: Nat Commun. 2022 Apr 21;13:2187. doi: 10.1038/s41467-022-29846-9 (PMC9023467; doi:10.1038/s41467-022-29846-9)
Supplement: Supplementary file 1 — Supplementary Information [file 41467_2022_29846_MOESM1_ESM.pdf]

SUPPLEMENTARY INFORMATION

USP22 regulates lipidome accumulation by stabilizing PPARγ in hepatocellular carcinoma

Ning et al.

Supplementary Table 1

Accession	Protein names	Gene names	MW [kDa]	Protein score	Sequence coverage (%)	# Unique Peptides	# Peptides	# PSMs
Q9UPT9	Ubiquitin carboxyl-terminal hydrolase 22	USP22	59.92	739.31	23.24	9	9	19
P37231	Peroxisome proliferator-activated receptor gamma	PPARG	57.58	598.64	30.5	11	11	13
P07814	Bifunctional glutamate/proline--tRNA ligase	EPRS	170.48	582.91	13.89	16	16	16
P40939	Trifunctional enzyme subunit alpha, mitochondrial	HADHA	82.95	552.95	21.36	11	11	12
P78527	DNA-dependent protein kinase catalytic subunit	PRKDC	468.79	545.18	4.65	16	16	16
P11940	Polyadenylate-binding protein 1	PABPC1	70.63	537.60	26.89	10	14	14
P41252	Isoleucine--tRNA ligase, cytoplasmic	IARS	144.41	536.24	14.98	13	13	14
P15924	Desmoplakin	DSP	331.57	519.46	5.75	10	10	11
O43795	Unconventional myosin-Ib	MYO1B	131.90	518.14	12.41	11	11	12
P52272	Heterogeneous nuclear ribonucleoprotein M	HNRNPM	77.46	505.45	17.4	9	9	10
P17987	T-complex protein 1 subunit alpha	TCP1	60.31	495.75	25.18	9	9	11
Q7Z406	Myosin-14	MYH14	227.73	491.72	5.11	3	7	10
P42704	eucine-rich PPR motif-containing protein, mitochondr	LRPPRC	157.81	491.62	9.18	10	10	10
Q6WCQ1	Myosin phosphatase Rho-interacting protein	MPRIP	116.46	484.18	17.07	11	11	11
P22314	Ubiquitin-like modifier-activating enzyme 1	UBA1	117.77	480.23	13.61	9	9	9
O00159	Unconventional myosin-Ic	MYO1C	121.61	470.75	10.35	9	9	11
O15020	Spectrin beta chain, non-erythrocytic 2	SPTBN2	271.16	464.62	6.28	8	10	10
P49411	Elongation factor Tu, mitochondrial	TUFM	49.51	455.61	28.76	10	10	11
P21333	Filamin-A	FLNA	280.56	453.28	7.86	14	14	14
P14923	Junction plakoglobin	JUP	81.69	452.28	19.6	12	12	14
Q00839	Heterogeneous nuclear ribonucleoprotein U	HNRNPU	90.53	447.26	12.97	8	8	11
Q9NR30	Nucleolar RNA helicase 2	DDX21	87.29	417.97	16.48	8	9	10
Q9ULV4	Coronin-1C	CORO1C	53.22	417.16	20.25	9	9	10
P11021	Endoplasmic reticulum chaperone BiP	HSPA5	72.29	414.13	18.96	7	8	9
P00558	Phosphoglycerate kinase 1	PGK1	44.59	411.79	25.9	10	10	10
Q9NZI8	Insulin-like growth factor 2 mRNA-binding protein 1	IGF2BP1	63.44	408.02	14.04	6	6	7
P25705	ATP synthase subunit alpha, mitochondrial	ATP5F1A	59.71	407.78	21.16	10	10	10
P04406	Glyceraldehyde-3-phosphate dehydrogenase	GAPDH	36.03	404.05	29.85	7	7	8
P13489	Ribonuclease inhibitor	RNH1	49.94	403.33	25.38	8	8	9
P17844	Probable ATP-dependent RNA helicase DDX5	DDX5	69.10	389.13	18.08	5	8	9
Q04695	Keratin, type I cytoskeletal 17	KRT17	48.08	375.66	23.15	4	10	10
P19338	Nucleolin	NCL	76.57	372.51	16.76	9	9	9
P63244	Receptor of activated protein C kinase 1	RACK1	35.05	361.48	29.97	7	7	8
P62701	40S ribosomal protein S4, X isoform	RPS4X	29.58	354.87	34.6	8	8	11
P05388	60S acidic ribosomal protein P0	RPLP0	34.25	353.89	36.91	8	8	9
Q9P0K7	Ankycorbin	RAI14	109.97	348.40	10.92	6	6	6
Q96PK6	RNA-binding protein 14	RBM14	69.45	342.74	13	7	7	7
P04899	uanine nucleotide-binding protein G(i) subunit alpha-	GNAI2	40.43	341.35	32.11	5	8	8
Q9UHB6	LIM domain and actin-binding protein 1	LIMA1	85.17	339.84	13.97	8	8	9
P35221	Catenin alpha-1	CTNNA1	100.01	338.27	9.93	7	7	8
P61978	Heterogeneous nuclear ribonucleoprotein K	HNRNPK	50.94	337.79	20.09	6	6	6
P23396	40S ribosomal protein S3	RPS3	26.67	325.57	35.39	8	8	9
P14625	Endoplasmin	HSP90B1	92.41	322.15	12.2	6	7	7
P27708	CAD protein	CAD	242.83	317.27	4.63	7	7	7
O15523	ATP-dependent RNA helicase DDX3Y	DDX3Y	73.11	316.38	12.42	6	7	8
Q92841	Probable ATP-dependent RNA helicase DDX17	DDX17	80.22	314.82	10.56	3	6	7
P13639	Elongation factor 2	EEF2	95.28	311.70	11.77	8	8	8
P08754	Guanine nucleotide-binding protein G(k) subunit alpha	GNAI3	40.51	309.00	24.86	4	7	7
P0DP24	Calmodulin-2	CALM2	16.83	307.97	42.28	5	5	6
P50991	T-complex protein 1 subunit delta	CCT4	57.89	307.20	20.41	8	8	8
P31943	Heterogeneous nuclear ribonucleoprotein H	HNRNPH1	49.20	304.18	22.72	6	7	8
P78371	T-complex protein 1 subunit beta	CCT2	57.45	302.87	19.44	6	6	6
Q02413	Desmoglein-1	DSG1	113.68	301.13	5.82	4	4	5
P08708	40S ribosomal protein S17	RPS17	15.54	300.19	40	4	4	5
P63104	14-3-3 protein zeta/delta	YWHAZ	27.73	299.87	29.8	4	5	5

Q12905	Interleukin enhancer-binding factor 2	ILF2	43.04	294.14	21.28	5	5	6
P05787	Keratin, type II cytoskeletal 8	KRT8	53.67	293.78	12.42	4	7	9
P62873	ne nucleotide-binding protein G(I)/G(S)/G(T) subunit	GNB1	37.35	293.77	23.53	3	7	7
P12277	Creatine kinase B-type	CKB	42.62	292.65	25.46	6	6	6
P62258	14-3-3 protein epsilon	YWHAE	29.16	292.57	29.8	5	6	7
P04075	Fructose-bisphosphate aldolase A	ALDOA	39.40	290.02	26.65	6	6	7
P11586	C-1-tetrahydrofolate synthase, cytoplasmic	MTHFD1	101.50	289.38	8.98	7	7	7
Q8NCA5	Protein FAM98A	FAM98A	55.37	288.87	10.21	3	3	4
P62753	40S ribosomal protein S6	RPS6	28.66	288.34	18.88	4	4	5
P62263	40S ribosomal protein S14	RPS14	16.26	284.62	29.8	4	4	5
P36578	60S ribosomal protein L4	RPL4	47.67	283.44	24.82	8	8	8
P46940	Ras GTPase-activating-like protein IQGAP1	IQGAP1	189.13	277.95	5.91	7	7	8
O75643	U5 small nuclear ribonucleoprotein 200 kDa helicase	SNRNP200	244.35	276.18	3.28	5	5	5
Q12923	Tyrosine-protein phosphatase non-receptor type 13	PTPN13	276.73	276.05	3.82	7	7	7
P46777	60S ribosomal protein L5	RPL5	34.34	273.20	20.88	5	5	7
Q9Y265	RuvB-like 1	RUVBL1	50.20	266.20	19.96	6	6	6
Q99832	T-complex protein 1 subunit eta	CCT7	59.33	263.91	12.52	4	4	5
P50990	T-complex protein 1 subunit theta	CCT8	59.58	262.74	12.96	6	6	6
P46087	bable 28S rRNA (cytosine(4447)-C(5))-methyltransfe	NOP2	89.25	261.39	9.85	7	7	7
Q14254	Flotillin-2	FLOT2	47.03	260.27	17.99	6	6	6
P63096	uanine nucleotide-binding protein G(i) subunit alpha-	GNAI1	40.34	259.08	19.21	3	6	6
P62241	40S ribosomal protein S8	RPS8	24.19	255.70	26.92	5	5	6
Q04637	Eukaryotic translation initiation factor 4 gamma 1	EIF4G1	175.38	255.49	4.5	6	6	6
Q08211	ATP-dependent RNA helicase A	DHX9	140.87	255.22	5.59	5	5	5
Q13310	Polyadenylate-binding protein 4	PABPC4	70.74	252.83	11.02	1	5	5
Q12931	Heat shock protein 75 kDa, mitochondrial	TRAP1	80.06	252.01	6.96	2	3	4
P61247	40S ribosomal protein S3a	RPS3A	29.93	249.28	24.24	6	6	7
P62879	ne nucleotide-binding protein G(I)/G(S)/G(T) subunit	GNB2	37.31	249.14	25.59	1	7	7
O75955	Flotillin-1	FLOT1	47.33	246.66	15.46	5	5	5
P06576	ATP synthase subunit beta, mitochondrial	ATP5F1B	56.52	242.53	11.15	4	4	5
P54136	Arginine--tRNA ligase, cytoplasmic	RARS	75.33	236.49	12.42	6	6	7
P36957	cinyltransferase component of 2-oxoglutarate dehydro	DLST	48.72	234.52	16.56	6	6	7
O75369	Filamin-B	FLNB	277.99	233.79	3.34	6	6	6
P62424	60S ribosomal protein L7a	RPL7A	29.98	233.36	14.66	4	4	5
Q15366	Poly(rC)-binding protein 2	PCBP2	38.56	233.24	23.29	4	6	6
P22626	Heterogeneous nuclear ribonucleoproteins A2/B1	HNRNPA2B1	37.41	232.16	17.28	5	6	7
Q12906	Interleukin enhancer-binding factor 3	ILF3	95.28	229.69	5.93	4	4	4
P46782	40S ribosomal protein S5	RPS5	22.86	226.61	25.49	3	3	4
Q4VCS5	Angiomotin	AMOT	118.01	224.72	6.37	4	4	4
P34932	Heat shock 70 kDa protein 4	HSPA4	94.27	224.49	6.9	4	4	4
P04181	Ornithine aminotransferase, mitochondrial	OAT	48.50	222.96	12.07	4	4	4
P26599	Polypyrimidine tract-binding protein 1	PTBP1	57.19	221.49	15.63	4	4	4
O43491	Band 4.1-like protein 2	EPB41L2	112.52	220.96	9.15	5	6	6
Q8WWI1	LIM domain only protein 7	LMO7	192.58	219.75	4.28	5	5	5
P62829	60S ribosomal protein L23	RPL23	14.86	217.02	25	2	2	5
Q14697	Neutral alpha-glucosidase AB	GANAB	106.81	215.62	4.77	4	4	4
P00338	L-lactate dehydrogenase A chain	LDHA	36.67	212.90	12.35	2	4	4
O14974	Protein phosphatase 1 regulatory subunit 12A	PPP1R12A	115.21	211.23	6.41	5	5	5
O94832	Unconventional myosin-Id	MYO1D	116.13	211.00	4.57	4	4	4
P68104	Elongation factor 1-alpha 1	EEF1A1	50.11	209.47	13.85	3	3	5
P09972	Fructose-bisphosphate aldolase C	ALDOC	39.43	207.47	6.32	1	1	3
P49368	T-complex protein 1 subunit gamma	CCT3	60.50	206.69	12.66	5	5	5
P13010	X-ray repair cross-complementing protein 5	XRCC5	82.65	205.96	10.66	5	5	5
P14868	Aspartate--tRNA ligase, cytoplasmic	DARS	57.10	205.85	12.18	5	5	5
O95831	Apoptosis-inducing factor 1, mitochondrial	AIFM1	66.86	204.90	10.6	5	5	5
Q8N1N4	Keratin, type II cytoskeletal 78	KRT78	56.83	204.06	11.73	5	6	6
P50402	Emerin	EMD	28.98	202.59	17.32	3	3	3
P04843	diphosphooligosaccharide--protein glycosyltransferase	RPN1	68.53	202.53	10.05	5	5	5

P52701	DNA mismatch repair protein Msh6	MSH6	152.69	200.81	4.93	5	5	5
Q9NNW5	WD repeat-containing protein 6	WDR6	121.65	200.17	6.6	4	4	4
O43390	Heterogeneous nuclear ribonucleoprotein R	HNRNPR	70.90	198.19	6	2	3	3
Q07065	Cytoskeleton-associated protein 4	CKAP4	65.98	196.26	8.8	4	4	4
P26373	60S ribosomal protein L13	RPL13	24.25	196.06	22.75	5	5	6
P38646	Stress-70 protein, mitochondrial	HSPA9	73.63	195.19	7.22	4	4	4
P12956	X-ray repair cross-complementing protein 6	XRCC6	69.80	193.70	8.37	4	4	4
Q00610	Clathrin heavy chain 1	CLTC	191.49	191.27	3.82	6	6	6
P12236	ADP/ATP translocase 3	SLC25A6	32.85	190.94	16.11	2	5	6
P05141	ADP/ATP translocase 2	SLC25A5	32.83	190.02	16.11	2	5	6
Q9HAV0	Guanine nucleotide-binding protein subunit beta-4	GNB4	37.54	189.61	16.76	1	6	6
Q9Y2J2	Band 4.1-like protein 3	EPB41L3	120.60	189.31	5.61	4	5	5
P61981	14-3-3 protein gamma	YWHAG	28.28	183.99	21.46	3	4	4
P05023	odium/potassium-transporting ATPase subunit alpha-	ATP1A1	112.82	183.84	4.59	3	3	3
P35222	Catenin beta-1	CTNNB1	85.44	183.50	4.99	3	3	3
P62917	60S ribosomal protein L8	RPL8	28.01	181.83	13.23	3	3	4
P13646	Keratin, type I cytoskeletal 13	KRT13	49.56	181.40	9.83	1	4	4
P62805	Histone H4	HIST1H4A	11.36	181.36	33.01	3	3	4
P13667	Protein disulfide-isomerase A4	PDIA4	72.89	181.14	9.77	4	4	4
P52907	F-actin-capping protein subunit alpha-1	CAPZA1	32.90	180.52	23.08	4	4	4
P18124	60S ribosomal protein L7	RPL7	29.21	180.47	26.61	6	6	6
Q16891	MICOS complex subunit MIC60	IMMT	83.63	179.96	9.23	5	5	5
P27635	60S ribosomal protein L10	RPL10	24.59	177.75	10.28	2	2	3
P30050	60S ribosomal protein L12	RPL12	17.81	177.05	24.85	3	3	3
P62851	40S ribosomal protein S25	RPS25	13.73	174.44	24	4	4	4
P39019	40S ribosomal protein S19	RPS19	16.05	174.09	26.9	4	4	4
Q9NX63	MICOS complex subunit MIC19	CHCHD3	26.14	173.28	15.42	3	3	3
Q99623	Prohibitin-2	PHB2	33.28	172.52	15.38	4	4	4
O60884	DnaJ homolog subfamily A member 2	DNAJA2	45.72	171.31	15.05	4	4	4
O14929	Histone acetyltransferase type B catalytic subunit	HAT1	49.48	169.51	9.79	3	3	3
P60842	Eukaryotic initiation factor 4A-I	EIF4A1	46.12	169.35	14.29	4	4	4
Q13263	Transcription intermediary factor 1-beta	TRIM28	88.49	167.29	8.74	4	4	4
Q9Y3I0	tRNA-splicing ligase RtcB homolog	RTCB	55.17	167.23	12.48	5	5	5
P51991	Heterogeneous nuclear ribonucleoprotein A3	HNRNPA3	39.57	166.50	10.05	2	3	4
P07910	Heterogeneous nuclear ribonucleoproteins C1/C2	HNRNPC	33.65	166.43	15.69	4	4	4
P23528	Cofilin-1	CFL1	18.49	166.25	34.34	4	4	4
P39023	60S ribosomal protein L3	RPL3	46.08	164.94	14.14	4	4	4
P63092	e nucleotide-binding protein G(s) subunit alpha isofor	GNAS	45.64	164.90	11.93	3	4	4
P40227	T-complex protein 1 subunit zeta	CCT6A	57.99	161.36	12.99	4	4	4
P51116	'ragile X mental retardation syndrome-related protein	FXR2	74.18	160.34	6.84	2	4	4
P56192	Methionine--tRNA ligase, cytoplasmic	MARS	101.05	159.08	5.89	4	4	4
P02545	Prelamin-A/C	LMNA	74.09	157.66	6.63	3	3	3
Q14684	Ribosomal RNA processing protein 1 homolog B	RRP1B	84.38	155.89	8.31	4	4	4
P09651	Heterogeneous nuclear ribonucleoprotein A1	HNRNPA1	38.72	155.88	6.72	1	2	3
P47897	Glutamine--tRNA ligase	QARS	87.74	155.65	4.39	2	2	2
P40926	Malate dehydrogenase, mitochondrial	MDH2	35.48	154.62	13.31	3	3	3
P29401	Transketolase	TKT	67.83	153.79	6.58	2	2	3
Q07020	60S ribosomal protein L18	RPL18	21.62	153.11	13.83	2	2	2
Q07157	Tight junction protein ZO-1	TJP1	195.34	152.70	3.49	4	4	4
Q9P2J5	Leucine--tRNA ligase, cytoplasmic	LARS	134.38	152.47	3.49	3	3	3
O60506	Heterogeneous nuclear ribonucleoprotein Q	SYNCRIP	69.56	151.57	7.7	3	4	4
P62269	40S ribosomal protein S18	RPS18	17.71	151.11	24.34	4	4	4
Q14974	Importin subunit beta-1	KPNB1	97.11	150.56	5.48	4	4	4
P13797	Plastin-3	PLS3	70.77	150.25	7.3	4	4	4
P51114	'ragile X mental retardation syndrome-related protein	FXR1	69.68	150.06	7.57	2	4	4
P32969	60S ribosomal protein L9	RPL9	21.85	148.64	15.1	2	2	3
P55060	Exportin-2	CSE1L	110.35	147.64	2.47	2	2	3
Q14152	Eukaryotic translation initiation factor 3 subunit A	EIF3A	166.47	147.41	3.4	4	4	4

P46781	40S ribosomal protein S9	RPS9	22.58	145.17	25.26	5	5	5
Q92499	ATP-dependent RNA helicase DDX1	DDX1	82.38	144.84	8.51	4	4	4
O43143	RNA-splicing factor ATP-dependent RNA helicase D	DHX15	90.88	143.57	5.53	4	4	4
P84098	60S ribosomal protein L19	RPL19	23.45	143.39	13.27	2	2	2
Q99459	Cell division cycle 5-like protein	CDC5L	92.19	143.37	5.86	3	3	3
Q99700	Ataxin-2	ATXN2	140.20	142.40	2.36	2	2	2
Q14498	RNA-binding protein 39	RBM39	59.34	142.06	6.04	2	2	2
P29966	Myristoylated alanine-rich C-kinase substrate	MARCKS	31.54	141.67	15.36	2	2	3
P05783	Keratin, type I cytoskeletal 18	KRT18	48.03	141.07	9.07	3	3	3
P62888	60S ribosomal protein L30	RPL30	12.78	140.84	24.35	2	2	2
Q92928	Putative Ras-related protein Rab-1C	RAB1C	22.00	139.23	18.91	3	3	3
Q6P2Q9	Pre-mRNA-processing-splicing factor 8	PRPF8	273.43	139.08	1.71	4	4	4
O75487	Glypican-4	GPC4	62.37	138.65	5.76	2	2	2
P36873	/threonine-protein phosphatase PP1-gamma catalytic s	PPP1CC	36.96	138.10	8.36	2	2	3
P62910	60S ribosomal protein L32	RPL32	15.85	137.90	25.19	3	3	3
Q15365	Poly(rC)-binding protein 1	PCBP1	37.47	136.10	12.64	1	3	3
P30837	Aldehyde dehydrogenase X, mitochondrial	ALDH1B1	57.17	135.59	9.28	3	3	3
P22102	Trifunctional purine biosynthetic protein adenosine-3	GART	107.70	133.72	3.37	2	2	2
P62899	60S ribosomal protein L31	RPL31	14.45	133.70	24	3	3	3
Q86VP6	Cullin-associated NEDD8-dissociated protein 1	CAND1	136.29	132.40	3.5	4	4	4
O00459	Phosphatidylinositol 3-kinase regulatory subunit beta	PIK3R2	81.49	132.14	6.87	3	3	3
Q02543	60S ribosomal protein L18a	RPL18A	20.75	131.93	19.89	3	3	3
Q8WXF1	Paraspeckle component 1	PSPC1	58.71	130.36	6.5	2	2	2
P20042	Eukaryotic translation initiation factor 2 subunit 2	EIF2S2	38.36	128.57	10.81	3	3	3
O00425	Insulin-like growth factor 2 mRNA-binding protein 3	IGF2BP3	63.67	127.78	4.66	2	2	2
Q96KK5	Histone H2A type 1-H	HIST1H2AH	13.90	127.52	21.88	2	2	3
P62913	60S ribosomal protein L11	RPL11	20.24	127.21	19.66	3	3	3
P38159	RNA-binding motif protein, X chromosome	RBMX	42.31	126.92	9.46	3	3	3
P31939	Bifunctional purine biosynthesis protein PURH	ATIC	64.58	126.71	6.42	2	2	2
P78347	General transcription factor II-I	GTF2I	112.35	126.66	4.01	3	3	3
P61204	ADP-ribosylation factor 3	ARF3	20.59	125.62	22.1	3	3	3
P34897	Serine hydroxymethyltransferase, mitochondrial	SHMT2	55.96	125.31	7.14	3	3	3
P52597	Heterogeneous nuclear ribonucleoprotein F	HNRNPF	45.64	124.83	12.05	2	3	3
O95373	Importin-7	IPO7	119.44	124.78	3.56	3	3	3
P40429	60S ribosomal protein L13a	RPL13A	23.56	122.89	16.75	3	3	3
A5YKK6	CCR4-NOT transcription complex subunit 1	CNOT1	266.77	122.33	1.43	3	3	3
P60174	Triosephosphate isomerase	TPI1	30.77	122.00	12.24	3	3	3
P45880	Voltage-dependent anion-selective channel protein 2	VDAC2	31.55	121.92	11.9	3	3	3
P17812	CTP synthase 1	CTPS1	66.65	121.51	5.58	3	3	3
P62750	60S ribosomal protein L23a	RPL23A	17.68	121.40	20.51	3	3	3
P47756	F-actin-capping protein subunit beta	CAPZB	31.33	119.67	13	3	3	3
Q9UN86	Ras GTPase-activating protein-binding protein 2	G3BP2	54.09	119.29	6.02	2	2	3
P11171	Protein 4.1	EPB41	96.96	118.36	5.67	3	3	3
Q9NZT1	Calmodulin-like protein 5	CALML5	15.88	118.25	25.34	3	3	3
Q2M1P5	Kinesin-like protein KIF7	KIF7	150.49	117.87	2.98	3	3	3
P06744	Glucose-6-phosphate isomerase	GPI	63.11	117.36	4.66	2	2	2
Q92485	Acid sphingomyelinase-like phosphodiesterase 3b	SMPDL3B	50.78	117.08	4.84	2	2	2
O15355	Protein phosphatase 1G	PPM1G	59.23	116.57	5.13	2	2	2
Q06787	Synaptic functional regulator FMR1	FMR1	71.13	114.85	3.32	1	2	2
P27348	14-3-3 protein theta	YWHAQ	27.75	114.64	9.8	1	2	2
P62249	40S ribosomal protein S16	RPS16	16.44	114.63	19.18	3	3	3
Q9P2E9	Ribosome-binding protein 1	RRBP1	152.36	114.12	3.26	2	2	2
Q9Y3F4	Serine-threonine kinase receptor-associated protein	STRAP	38.41	113.11	8.86	2	2	2
Q16643	Drebrin	DBN1	71.39	111.83	5.24	2	2	2
Q9UQ80	Proliferation-associated protein 2G4	PA2G4	43.76	110.98	5.84	2	2	2
P04844	diphosphooligosaccharide--protein glycosyltransferase	RPN2	69.24	110.57	5.23	2	2	2
P42167	Lamina-associated polypeptide 2, isoforms beta/gamm	TMPO	50.64	110.35	12.11	3	3	3
P36542	ATP synthase subunit gamma, mitochondrial	ATP5F1C	32.98	109.65	7.38	2	2	2

P46779	60S ribosomal protein L28	RPL28	15.74	109.47	16.79	3	3	3
P62826	GTP-binding nuclear protein Ran	RAN	24.41	109.14	15.74	3	3	3
P32119	Peroxiredoxin-2	PRDX2	21.88	108.89	18.18	2	3	3
P60866	40S ribosomal protein S20	RPS20	13.36	108.70	9.24	1	1	2
P46776	60S ribosomal protein L27a	RPL27A	16.55	108.20	15.54	2	2	2
P30101	Protein disulfide-isomerase A3	PDIA3	56.75	107.61	7.33	3	3	3
P23526	Adenosylhomocysteinase	AHCY	47.69	107.45	8.1	3	3	3
Q9UBI6	nucleotide-binding protein G(I)/G(S)/G(O) subunit ga	GNG12	8.00	107.43	56.94	3	3	3
P31025	Lipocalin-1	LCN1	19.24	106.57	12.5	2	2	2
P35268	60S ribosomal protein L22	RPL22	14.78	106.52	18.75	2	2	2
Q14318	Peptidyl-prolyl cis-trans isomerase FKBP8	FKBP8	44.53	106.11	3.88	1	1	1
Q02878	60S ribosomal protein L6	RPL6	32.71	105.91	11.46	3	3	3
P32322	Pyrroline-5-carboxylate reductase 1, mitochondrial	PYCR1	33.34	105.23	8.78	2	2	2
Q92616	eIF-2-alpha kinase activator GCN1	GCN1	292.57	104.86	1.57	3	3	3
P83731	60S ribosomal protein L24	RPL24	17.77	104.82	13.38	2	2	2
Q9UQ35	Serine/arginine repetitive matrix protein 2	SRRM2	299.44	104.62	1.53	3	3	3
P06748	Nucleophosmin	NPM1	32.55	104.19	6.8	2	2	2
Q9BUJ2	eterogeneous nuclear ribonucleoprotein U-like protein	HNRNPUL1	95.68	103.75	3.97	2	2	2
P62906	60S ribosomal protein L10a	RPL10A	24.82	103.41	19.35	3	3	3
P05455	Lupus La protein	SSB	46.81	103.21	7.6	3	3	3
Q13573	SNW domain-containing protein 1	SNW1	61.46	102.89	6.9	2	2	2
P60468	Protein transport protein Sec61 subunit beta	SEC61B	9.97	102.78	26.04	2	2	2
P06241	Tyrosine-protein kinase Fyn	FYN	60.72	102.23	5.4	2	3	3
P54886	Delta-1-pyrroline-5-carboxylate synthase	ALDH18A1	87.25	102.22	4.03	2	3	3
P13674	Prolyl 4-hydroxylase subunit alpha-1	P4HA1	61.01	102.07	4.87	2	2	2
P42677	40S ribosomal protein S27	RPS27	9.45	101.12	25	2	2	2
Q14344	Guanine nucleotide-binding protein subunit alpha-13	GNA13	44.02	100.39	7.43	1	2	2
P46783	40S ribosomal protein S10	RPS10	18.89	100.13	9.09	2	2	2
Q9Y285	Phenylalanine--tRNA ligase alpha subunit	FARSA	57.53	99.81	4.33	2	2	2
O14744	Protein arginine N-methyltransferase 5	PRMT5	72.64	99.53	2.04	1	1	1
Q8N1F7	Nuclear pore complex protein Nup93	NUP93	93.43	99.41	4.88	3	3	3
Q06830	Peroxiredoxin-1	PRDX1	22.10	99.20	17.59	2	3	3
Q92522	Histone H1x	H1FX	22.47	98.33	12.68	2	2	2
P55072	Transitional endoplasmic reticulum ATPase	VCP	89.27	97.08	2.48	1	1	1
P61513	60S ribosomal protein L37a	RPL37A	10.27	96.40	19.57	1	1	1
Q5T749	Keratinocyte proline-rich protein	KPRP	64.09	95.91	7.6	3	3	3
Q16181	Septin-7	SEPT7	50.65	95.79	5.26	2	2	3
P61221	ATP-binding cassette sub-family E member 1	ABCE1	67.27	95.57	4.34	2	2	2
Q96IX5	Up-regulated during skeletal muscle growth protein 5	USMG5	6.45	95.23	25.86	1	1	1
Q9BYG3	KI67 FHA domain-interacting nucleolar phosphoprot	NIFK	34.20	95.19	7.85	1	1	1
Q13283	Ras GTPase-activating protein-binding protein 1	G3BP1	52.13	94.82	7.3	2	2	2
Q02978	Mitochondrial 2-oxoglutarate/malate carrier protein	SLC25A11	34.04	94.81	5.1	1	1	1
P57088	Transmembrane protein 33	TMEM33	27.96	94.69	8.91	2	2	2
Q96HR8	ACA ribonucleoprotein complex non-core subunit NA	NAF1	53.68	93.50	3.04	1	1	1
Q14103	Heterogeneous nuclear ribonucleoprotein D0	HNRNPD	38.41	93.34	9.3	2	2	2
Q14203	Dynactin subunit 1	DCTN1	141.61	92.34	1.88	2	2	2
P09471	uanine nucleotide-binding protein G(o) subunit alph	GNAO1	40.02	90.78	7.34	1	2	2
P14678	all nuclear ribonucleoprotein-associated proteins B an	SNRPB	24.59	90.57	9.17	2	2	2
O60762	Dolichol-phosphate mannosyltransferase subunit 1	DPM1	29.62	90.55	8.46	2	2	2
Q3ZCQ8	hondrial import inner membrane translocase subunit T	TIMM50	39.62	90.45	4.53	1	1	1
Q01780	Exosome component 10	EXOSC10	100.77	90.02	2.49	2	2	2
P60660	Myosin light polypeptide 6	MYL6	16.92	89.75	15.89	2	2	2
O14980	Exportin-1	XPO1	123.31	89.69	1.4	1	1	1
Q9Y3U8	60S ribosomal protein L36	RPL36	12.25	89.46	20	2	2	2
Q9Y5M8	Signal recognition particle receptor subunit beta	SRPRB	29.68	88.52	7.01	1	1	1
Q6PKG0	La-related protein 1	LARP1	123.43	88.45	2.28	1	1	1
Q9HB71	Calcyclin-binding protein	CACYBP	26.19	88.05	13.16	2	2	2
P18669	Phosphoglycerate mutase 1	PGAM1	28.79	87.84	11.81	2	2	2

Q52LJ0	Protein FAM98B	FAM98B	37.17	87.63	3.94	1	1	1
P15880	40S ribosomal protein S2	RPS2	31.30	87.21	9.9	3	3	3
Q5H9R7	rine/threonine-protein phosphatase 6 regulatory subun	PPP6R3	97.61	86.79	2.75	2	2	2
P41091	Eukaryotic translation initiation factor 2 subunit 3	EIF2S3	51.08	86.66	6.36	2	2	2
Q04837	Single-stranded DNA-binding protein, mitochondrial	SSBP1	17.25	86.57	16.22	2	2	2
Q12792	Twinfilin-1	TWF1	40.26	86.08	3.71	1	1	1
P07948	Tyrosine-protein kinase Lyn	LYN	58.54	85.81	6.05	2	3	3
Q13151	Heterogeneous nuclear ribonucleoprotein A0	HNRNPA0	30.82	85.76	6.56	1	2	3
P26368	Splicing factor U2AF 65 kDa subunit	U2AF2	53.47	85.54	4.42	1	1	2
P46778	60S ribosomal protein L21	RPL21	18.55	85.50	9.38	1	1	1
Q9BQG0	Myb-binding protein 1A	MYBBP1A	148.76	85.02	2.18	3	3	3
Q9Y490	Talin-1	TLN1	269.60	84.81	1.61	3	3	3
Q9BQ39	ATP-dependent RNA helicase DDX50	DDX50	82.51	84.45	3.12	1	2	2
Q86YZ3	Hornerin	HRNR	282.23	84.43	2.35	2	2	2
P10768	S-formylglutathione hydrolase	ESD	31.44	83.80	10.64	2	2	2
P52292	Importin subunit alpha-1	KPNA2	57.83	83.58	2.84	1	1	1
Q13155	yl tRNA synthase complex-interacting multifunctional	AIMP2	35.33	83.30	7.81	1	1	1
Q9H4G4	Golgi-associated plant pathogenesis-related protein 1	GLIPR2	17.21	82.72	16.88	2	2	2
P52294	Importin subunit alpha-5	KPNA1	60.18	82.58	3.35	1	1	1
P12268	Inosine-5'-monophosphate dehydrogenase 2	IMPDH2	55.77	82.39	4.47	2	2	2
Q8WWM7	Ataxin-2-like protein	ATXN2L	113.30	82.24	2.33	2	2	2
P04040	Catalase	CAT	59.72	82.24	4.55	2	2	2
P31153	S-adenosylmethionine synthase isoform type-2	MAT2A	43.63	82.18	3.8	1	1	1
P25205	DNA replication licensing factor MCM3	MCM3	90.92	81.84	3.09	2	2	2
Q99569	Plakophilin-4	PKP4	131.79	81.32	1.85	2	2	2
Q86UE4	Protein LYRIC	MTDH	63.80	80.90	3.95	2	2	2
P29992	Guanine nucleotide-binding protein subunit alpha-11	GNA11	42.10	80.85	5.29	2	2	2
Q96P63	Serpin B12	SERPINB12	46.25	80.78	6.42	2	2	2
P20700	Lamin-B1	LMNB1	66.37	80.61	4.1	2	2	2
Q99959	Plakophilin-2	PKP2	97.35	80.37	2.16	1	2	2
O00161	Synaptosomal-associated protein 23	SNAP23	23.34	80.32	11.37	2	2	2
P31689	DnaJ homolog subfamily A member 1	DNAJA1	44.84	79.91	4.79	2	2	2
P15531	Nucleoside diphosphate kinase A	NME1	17.14	79.21	20.39	2	2	2
P62280	40S ribosomal protein S11	RPS11	18.42	79.02	6.96	2	2	2
P00505	Aspartate aminotransferase, mitochondrial	GOT2	47.49	78.78	6.74	2	2	2
P62195	26S proteasome regulatory subunit 8	PSMC5	45.60	78.76	3.2	1	1	1
P62277	40S ribosomal protein S13	RPS13	17.21	78.63	8.61	2	2	2
O95232	Luc7-like protein 3	LUC7L3	51.44	78.42	4.17	1	1	1
Q00341	Vigilin	HDLBP	141.37	78.31	0.95	1	1	1
Q04917	14-3-3 protein eta	YWHAH	28.20	77.87	8.13	1	2	2
P16615	Sarcoplasmic/endoplasmic reticulum calcium ATPase	ATP2A2	114.68	77.86	1.44	1	1	1
Q6Y7W6	GRB10-interacting GYF protein 2	GIGYF2	149.98	77.84	2.69	2	2	2
P37108	Signal recognition particle 14 kDa protein	SRP14	14.56	77.83	10.29	1	1	1
Q13724	Mannosyl-oligosaccharide glucosidase	MOGS	91.86	77.45	2.15	1	1	1
P48634	Protein PRRC2A	PRRC2A	228.72	77.31	2.04	2	2	2
Q96AG4	Leucine-rich repeat-containing protein 59	LRRC59	34.91	77.10	12.05	2	2	2
O95757	Heat shock 70 kDa protein 4L	HSPA4L	94.45	76.78	1.67	1	1	1
P62244	40S ribosomal protein S15a	RPS15A	14.83	75.81	12.31	2	2	2
P60981	Destrin	DSTN	18.49	75.66	13.94	2	2	2
Q6UVY6	DBH-like monooxygenase protein 1	MOXD1	69.61	75.57	5.38	2	2	2
P62266	40S ribosomal protein S23	RPS23	15.80	75.02	13.29	2	2	2
O95292	sicle-associated membrane protein-associated protein	VAPB	27.21	74.66	4.94	1	1	1
P62081	40S ribosomal protein S7	RPS7	22.11	74.57	10.31	2	2	2
Q9P035	Very-long-chain (3R)-3-hydroxyacyl-CoA dehydratase	HACD3	43.13	74.56	3.87	1	1	1
O75477	Erlin-1	ERLIN1	38.90	74.23	7.51	2	2	2
A6NEC2	Puromycin-sensitive aminopeptidase-like protein	NPEPPSL1	53.71	73.95	6.28	2	2	2
Q16531	DNA damage-binding protein 1	DDB1	126.89	73.72	3.25	2	2	2
P11233	Ras-related protein Ral-A	RALA	23.55	73.63	6.8	1	1	1

P27986	Phosphatidylinositol 3-kinase regulatory subunit alpha	PIK3R1	83.55	73.53	1.93	1	1	1
P10155	60 kDa SS-A/Ro ribonucleoprotein	TROVE2	60.63	73.39	2.04	1	1	1
Q9UHB9	Signal recognition particle subunit SRP68	SRP68	70.69	73.32	4.31	2	2	2
P28288	ATP-binding cassette sub-family D member 3	ABCD3	75.43	73.07	2.28	1	2	2
P53621	Coatomer subunit alpha	COPA	138.26	72.77	1.31	1	1	1
P06493	Cyclin-dependent kinase 1	CDK1	34.07	72.70	7.41	1	2	2
O94887	RM, ARHGEF and pleckstrin domain-containing prote	FARP2	119.81	72.56	1.42	1	1	1
P04637	Cellular tumor antigen p53	TP53	43.63	72.54	3.31	1	1	1
Q15645	Pachytene checkpoint protein 2 homolog	TRIP13	48.52	72.03	3.01	1	1	1
Q12959	Disks large homolog 1	DLG1	100.39	71.84	1.33	1	1	1
P10599	Thioredoxin	TXN	11.73	71.77	12.38	1	1	1
Q5D862	Filaggrin-2	FLG2	247.93	71.77	0.5	1	1	1
P78346	Ribonuclease P protein subunit p30	RPP30	29.30	71.19	3.36	1	1	1
P16403	Histone H1.2	HIST1H1C	21.35	71.15	13.15	2	2	2
Q9Y3Y2	Chromatin target of PRMT1 protein	CHTOP	26.38	71.06	5.24	1	1	1
P62847	40S ribosomal protein S24	RPS24	15.41	70.70	11.28	1	1	1
O43324	Eukaryotic translation elongation factor 1 epsilon-1	EEF1E1	19.80	70.30	12.64	2	2	2
Q9ULH0	Kinase D-interacting substrate of 220 kDa	KIDINS220	196.42	69.95	1.19	1	1	1
P07737	Profilin-1	PFN1	15.04	69.55	11.43	1	1	1
P47914	60S ribosomal protein L29	RPL29	17.74	69.45	14.47	2	2	2
Q9Y520	Protein PRRC2C	PRRC2C	316.72	69.12	1.04	2	2	2
P26641	Elongation factor 1-gamma	EEF1G	50.09	68.98	5.95	2	2	2
P12270	Nucleoprotein TPR	TPR	267.13	67.88	0.42	1	1	1
Q9Y266	Nuclear migration protein nudC	NUDC	38.22	67.41	6.04	2	2	2
Q9HCE1	Putative helicase MOV-10	MOV10	113.60	67.40	2.49	2	2	2
Q7Z2W4	Zinc finger CCCH-type antiviral protein 1	ZC3HAV1	101.37	67.36	3.1	2	2	2
Q9Y383	Putative RNA-binding protein Luc7-like 2	LUC7L2	46.49	67.35	3.83	1	1	1
P24539	ATP synthase F(0) complex subunit B1, mitochondria	ATP5F1	28.89	67.34	4.69	1	1	1
Q9NRX1	RNA-binding protein PNO1	PNO1	27.91	67.01	6.35	1	1	1
O75533	Splicing factor 3B subunit 1	SF3B1	145.74	66.98	1.23	1	1	1
P61353	60S ribosomal protein L27	RPL27	15.79	66.82	14.71	2	2	2
Q14204	Cytoplasmic dynein 1 heavy chain 1	DYNC1H1	532.07	66.20	0.62	2	2	2
P35998	26S proteasome regulatory subunit 7	PSMC2	48.60	65.94	3	1	1	1
P08195	4F2 cell-surface antigen heavy chain	SLC3A2	67.95	65.79	2.06	1	1	1
P09960	Leukotriene A-4 hydrolase	LTA4H	69.24	64.82	2.13	1	1	1
Q9UEY8	Gamma-adducin	ADD3	79.11	64.57	1.98	1	1	1
Q08945	FACT complex subunit SSRP1	SSRP1	81.02	64.32	1.97	1	1	1
Q13200	26S proteasome non-ATPase regulatory subunit 2	PSMD2	100.14	63.89	2.64	2	2	2
P15153	Ras-related C3 botulinum toxin substrate 2	RAC2	21.42	63.66	5.21	1	1	1
Q5T9A4	ATPase family AAA domain-containing protein 3B	ATAD3B	72.53	63.39	3.55	2	2	2
O60814	Histone H2B type 1-K	HIST1H2BK	13.88	63.32	15.87	1	2	2
Q53GQ0	Very-long-chain 3-oxoacyl-CoA reductase	HSD17B12	34.30	62.99	4.49	1	1	1
P51648	Fatty aldehyde dehydrogenase	ALDH3A2	54.81	62.69	2.68	1	1	1
P07355	Annexin A2	ANXA2	38.58	62.30	5.01	1	1	1
Q96EP5	DAZ-associated protein 1	DAZAP1	43.36	62.21	3.69	1	1	1
Q92552	28S ribosomal protein S27, mitochondrial	MRPS27	47.58	62.15	3.62	1	1	1
O14828	Secretory carrier-associated membrane protein 3	SCAMP3	38.26	62.13	4.61	1	1	1
O00410	Importin-5	IPO5	123.55	62.02	1.37	1	1	1
Q86UK7	E3 ubiquitin-protein ligase ZNF598	ZNF598	98.58	61.77	1.33	1	1	1
Q15517	Corneodesmosin	CDSN	51.49	61.51	3.4	1	1	1
P43243	Matrin-3	MATR3	94.56	61.24	1.65	1	1	1
Q9BY77	Polymerase delta-interacting protein 3	POLDIP3	46.06	61.10	2.38	1	1	1
P49792	E3 SUMO-protein ligase RanBP2	RANBP2	357.97	60.74	0.47	1	1	1
P54577	Tyrosine--tRNA ligase, cytoplasmic	YARS	59.11	60.70	3.41	2	2	2
P10114	Ras-related protein Rap-2a	RAP2A	20.60	60.68	5.46	1	1	1
Q9UNS2	COP9 signalosome complex subunit 3	COPS3	47.84	60.22	2.84	1	1	1
Q96C36	Pyrroline-5-carboxylate reductase 2	PYCR2	33.62	60.16	5.31	1	1	1
Q16658	Fascin	FSCN1	54.50	59.99	2.64	1	1	1

P19086	Guanine nucleotide-binding protein G(z) subunit alpha	GNAZ	40.90	59.89	4.79	1	1	1
Q96QK1	Vacuolar protein sorting-associated protein 35	VPS35	91.65	59.84	1.76	1	1	1
O43423	c leucine-rich nuclear phosphoprotein 32 family mem	ANP32C	26.75	59.80	4.7	1	1	1
P81605	Dermcidin	DCD	11.28	59.63	10	1	1	1
P48643	T-complex protein 1 subunit epsilon	CCT5	59.63	59.50	3.7	1	1	1
Q8NEZ5	F-box only protein 22	FBXO22	44.48	59.35	3.97	1	1	1
P11908	Ribose-phosphate pyrophosphokinase 2	PRPS2	34.75	58.98	2.2	1	1	1
P27816	Microtubule-associated protein 4	MAP4	120.93	58.73	1.48	1	1	1
Q7L2E3	Putative ATP-dependent RNA helicase DHX30	DHX30	133.85	58.48	1.93	2	2	2
Q16881	Thioredoxin reductase 1, cytoplasmic	TXNRD1	70.86	58.46	1.69	1	1	1
Q9Y5J1	J3 small nucleolar RNA-associated protein 18 homolo	UTP18	61.96	58.28	2.34	1	1	1
P52565	Rho GDP-dissociation inhibitor 1	ARHGDIA	23.19	57.96	7.35	1	1	1
Q15758	Neutral amino acid transporter B(0)	SLC1A5	56.56	57.95	2.03	1	1	1
Q07021	nt component 1 Q subcomponent-binding protein, mi	C1QBP	31.34	57.84	4.61	1	1	1
P60900	Proteasome subunit alpha type-6	PSMA6	27.38	57.75	5.28	1	1	1
O95271	Tankyrase-1	TNKS	141.95	57.75	0.83	1	1	1
Q12797	Aspartyl/asparaginyl beta-hydroxylase	ASPH	85.81	57.55	2.37	1	1	1
P19105	Myosin regulatory light chain 12A	MYL12A	19.78	57.35	6.43	1	1	1
Q9UBM7	7-dehydrocholesterol reductase	DHCR7	54.45	57.31	4.63	1	1	1
Q9H307	Pinin	PNN	81.58	56.67	2.37	1	1	1
Q9Y2I1	Nischarin	NISCH	166.52	56.29	0.8	1	1	1
Q13601	KRR1 small subunit processome component homolog	KRR1	43.64	56.18	3.15	1	1	1
Q8IVF7	Formin-like protein 3	FMNL3	117.14	56.15	1.36	1	1	1
P17174	Aspartate aminotransferase, cytoplasmic	GOT1	46.22	56.14	3.39	1	1	1
O43592	Exportin-T	XPOT	109.89	55.88	1.25	1	1	1
Q96S44	EKC/KEOPS complex subunit TP53RK	TP53RK	28.14	55.81	8.3	1	1	1
Q9BR76	Coronin-1B	CORO1B	54.20	55.71	2.04	1	1	1
Q99575	Ribonucleases P/MRP protein subunit POP1	POP1	114.64	55.69	1.07	1	1	1
P50914	60S ribosomal protein L14	RPL14	23.42	55.60	5.12	1	1	1
Q96IZ0	PRKC apoptosis WT1 regulator protein	PAWR	36.55	55.57	3.82	1	1	1
P55884	Eukaryotic translation initiation factor 3 subunit B	EIF3B	92.42	55.41	1.72	1	1	1
Q9NTK5	Obg-like ATPase 1	OLA1	44.72	55.18	2.78	1	1	1
Q99613	Eukaryotic translation initiation factor 3 subunit C	EIF3C	105.28	55.18	1.1	1	1	1
O15371	Eukaryotic translation initiation factor 3 subunit D	EIF3D	63.93	54.71	2.19	1	1	1
Q00325	Phosphate carrier protein, mitochondrial	SLC25A3	40.07	54.61	3.31	1	1	1
P00387	NADH-cytochrome b5 reductase 3	CYB5R3	34.21	54.60	4.32	1	1	1
P0CG48	Polyubiquitin-C	UBC	76.99	54.32	21.02	1	1	1
O60841	Eukaryotic translation initiation factor 5B	EIF5B	138.74	54.18	1.15	1	1	1
P61313	60S ribosomal protein L15	RPL15	24.13	54.10	4.41	1	1	1
P18206	Vinculin	VCL	123.72	54.00	1.23	1	1	1
P02788	Lactotransferrin	LTF	78.13	53.96	1.41	1	1	1
O43790	Keratin, type II cuticular Hb6	KRT86	53.47	53.62	1.44	1	1	1
Q9UM54	Unconventional myosin-VI	MYO6	149.60	53.34	1.16	1	1	1
Q01469	Fatty acid-binding protein 5	FABP5	15.15	52.62	6.67	1	1	1
Q9NRP0	Oligosaccharyltransferase complex subunit OSTC	OSTC	16.82	52.61	8.05	1	1	1
P55209	Nucleosome assembly protein 1-like 1	NAP1L1	45.35	52.57	4.35	1	1	1
P62854	40S ribosomal protein S26	RPS26	13.01	51.91	13.04	1	1	1
Q6P2E9	Enhancer of mRNA-decapping protein 4	EDC4	151.57	51.85	1.43	1	1	1
Q08554	Desmocollin-1	DSC1	99.92	51.78	1.68	1	1	1
Q5M775	Cytospin-B	SPECC1	118.51	51.65	1.4	1	1	1
P18621	60S ribosomal protein L17	RPL17	21.38	51.16	5.43	1	1	1
P10515	acetyltransferase component of pyruvate dehydrogen	DLAT	68.95	51.16	1.39	1	1	1
O76094	Signal recognition particle subunit SRP72	SRP72	74.56	51.05	2.53	1	1	1
P49006	MARCKS-related protein	MARCKSL1	19.52	51.04	7.69	1	1	1
Q9Y2A7	Nck-associated protein 1	NCKAP1	128.71	50.99	1.42	1	1	1
O75821	Eukaryotic translation initiation factor 3 subunit G	EIF3G	35.59	50.90	4.38	1	1	1
P13804	ectron transfer flavoprotein subunit alpha, mitochondr	ETFA	35.06	50.81	5.71	1	1	1
Q10570	leavage and polyadenylation specificity factor subunit	CPSF1	160.78	50.80	1.18	1	1	1

P62937	Peptidyl-prolyl cis-trans isomerase A	PPIA	18.00	50.69	5.45	1	1	1
P46977	nosphooligosaccharide--protein glycosyltransferase su	STT3A	80.48	50.59	1.28	1	1	1
P05387	60S acidic ribosomal protein P2	RPLP2	11.66	50.50	10.43	1	1	1
P49915	GMP synthase [glutamine-hydrolyzing]	GMPS	76.67	50.26	1.44	1	1	1
P48444	Coatomer subunit delta	ARCN1	57.17	50.26	2.15	1	1	1
O60488	Long-chain-fatty-acid--CoA ligase 4	ACSL4	79.14	50.13	2.53	1	1	1
Q5T3I0	G patch domain-containing protein 4	GPATCH4	50.35	50.01	2.47	1	1	1
P49207	60S ribosomal protein L34	RPL34	13.28	49.99	7.69	1	1	1
Q9NYF8	Bcl-2-associated transcription factor 1	BCLAF1	106.06	49.95	1.2	1	1	1
Q99729	Heterogeneous nuclear ribonucleoprotein A/B	HNRNPAB	36.20	49.67	3.92	1	1	1
P30153	e-protein phosphatase 2A 65 kDa regulatory subunit A	PPP2R1A	65.27	49.41	1.7	1	1	1
O00231	26S proteasome non-ATPase regulatory subunit 11	PSMD11	47.43	49.32	2.37	1	1	1
P61626	Lysozyme C	LYZ	16.53	49.04	18.92	1	1	1
Q7L576	Cytoplasmic FMR1-interacting protein 1	CYFIP1	145.09	49.02	0.88	1	1	1
P42766	60S ribosomal protein L35	RPL35	14.54	48.97	8.13	1	1	1
P46060	Ran GTPase-activating protein 1	RANGAP1	63.50	48.84	2.04	1	1	1
P08243	Asparagine synthetase [glutamine-hydrolyzing]	ASNS	64.33	48.69	1.96	1	1	1
O60716	Catenin delta-1	CTNND1	108.10	48.58	0.72	1	1	1
Q8N0X7	Spartin	SPART	72.79	48.46	1.8	1	1	1
Q9NVP1	ATP-dependent RNA helicase DDX18	DDX18	75.36	48.44	1.64	1	1	1
P51654	Glypican-3	GPC3	65.52	48.30	2.07	1	1	1
P31944	Caspase-14	CASP14	27.66	48.22	5.37	1	1	1
Q9UG63	ATP-binding cassette sub-family F member 2	ABCF2	71.24	48.19	2.25	1	1	1
Q9Y230	RuvB-like 2	RUVBL2	51.12	48.14	3.67	1	1	1
P09661	U2 small nuclear ribonucleoprotein A'	SNRPA1	28.40	47.86	6.67	1	1	1
O76021	Ribosomal L1 domain-containing protein 1	RSL1D1	54.94	47.82	2.04	1	1	1
P62495	Eukaryotic peptide chain release factor subunit 1	ETF1	49.00	47.79	1.6	1	1	1
P51571	Translocon-associated protein subunit delta	SSR4	18.99	47.32	7.51	1	1	1
P62070	Ras-related protein R-Ras2	RRAS2	23.38	46.69	5.88	1	1	1
P62318	Small nuclear ribonucleoprotein Sm D3	SNRPD3	13.91	46.56	7.94	1	1	1
Q8WZ42	Titin	TTN	3813.65	46.56	0.02	1	1	1
Q9BXJ9	N-alpha-acetyltransferase 15, NatA auxiliary subunit	NAA15	101.21	46.48	1.04	1	1	1
P63218	nucleotide-binding protein G(I)/G(S)/G(O) subunit g	GNG5	7.31	46.45	13.24	1	1	1
Q96SB4	SRSF protein kinase 1	SRPK1	74.28	46.27	1.53	1	1	1
O76009	Keratin, type I cuticular Ha3-I	KRT33A	45.91	46.25	3.22	1	1	1
Q6KB66	Keratin, type II cytoskeletal 80	KRT80	50.49	46.24	2.65	1	1	1
Q9Y4Z0	U6 snRNA-associated Sm-like protein LSm4	LSM4	15.34	46.20	5.04	1	1	1
O75534	Cold shock domain-containing protein E1	CSDE1	88.83	46.15	1.38	1	1	1
O43684	Mitotic checkpoint protein BUB3	BUB3	37.13	46.11	3.05	1	1	1
Q9H0A0	RNA cytidine acetyltransferase	NAT10	115.66	46.11	1.07	1	1	1
P59998	Actin-related protein 2/3 complex subunit 4	ARPC4	19.65	45.81	6.55	1	1	1
Q9NZ01	Very-long-chain enoyl-CoA reductase	TECR	36.01	45.77	3.9	1	1	1
P62861	40S ribosomal protein S30	FAU	6.64	45.62	16.95	1	1	1
P48047	ATP synthase subunit O, mitochondrial	ATP5O	23.26	45.55	5.16	1	1	1
P61163	Alpha-centractin	ACTR1A	42.59	45.44	5.85	1	1	1
P26196	Probable ATP-dependent RNA helicase DDX6	DDX6	54.38	45.43	1.24	1	1	1
Q9HAC8	Ubiquitin domain-containing protein 1	UBTD1	25.92	45.31	5.73	1	1	1
Q7KZF4	Staphylococcal nuclease domain-containing protein 1	SND1	101.93	45.28	1.76	1	1	1
Q6P1J9	Parafibromin	CDC73	60.54	45.26	1.51	1	1	1
P35232	Prohibitin	PHB	29.79	44.96	4.04	1	1	1
P62841	40S ribosomal protein S15	RPS15	17.03	44.93	8.28	1	1	1
A0A0J9YX35	Immunoglobulin heavy variable 3-64D	IGHV3-64D	12.81	44.89	9.4	1	1	1
Q9Y5A9	YTH domain-containing family protein 2	YTHDF2	62.30	44.78	1.9	1	1	1
O43670	3-interacting and GLEBS motif-containing protein ZN	ZNF207	50.72	44.59	2.51	1	1	1
P35520	Cystathionine beta-synthase	CBS	60.55	44.56	1.27	1	1	1
P40925	Malate dehydrogenase, cytoplasmic	MDH1	36.40	44.53	3.89	1	1	1
P62316	Small nuclear ribonucleoprotein Sm D2	SNRPD2	13.52	44.33	5.93	1	1	1
P00367	Glutamate dehydrogenase 1, mitochondrial	GLUD1	61.36	44.23	1.97	1	1	1

Q8IY81	pre-rRNA processing protein FTSJ3	FTSJ3	96.50	44.22	2.83	1	1	1
Q9BTT6	Leucine-rich repeat-containing protein 1	LRRC1	59.20	44.20	1.91	1	1	1
Q00688	Peptidyl-prolyl cis-trans isomerase FKBP3	FKBP3	25.16	44.19	4.91	1	1	1
O14818	Proteasome subunit alpha type-7	PSMA7	27.87	44.18	4.44	1	1	1
P22234	Multifunctional protein ADE2	PAICS	47.05	44.17	2.35	1	1	1
P28482	Mitogen-activated protein kinase 1	MAPK1	41.36	44.04	1.94	1	1	1
Q13243	Serine/arginine-rich splicing factor 5	SRSF5	31.25	43.92	4.41	1	1	1
Q9Y4P3	Transducin beta-like protein 2	TBL2	49.77	43.65	2.91	1	1	1
Q9BRJ6	Uncharacterized protein C7orf50	C7orf50	22.07	43.38	11.34	1	1	1
P61254	60S ribosomal protein L26	RPL26	17.25	43.37	6.21	1	1	1
O75352	Mannose-P-dolichol utilization defect 1 protein	MPDU1	26.62	43.21	4.05	1	1	1
Q8NI36	WD repeat-containing protein 36	WDR36	105.26	43.19	1.58	1	1	1
Q01105	Protein SET	SET	33.47	43.13	3.79	1	1	1
Q15149	Plectin	PLEC	531.47	42.99	0.19	1	1	1
P63151	e-protein phosphatase 2A 55 kDa regulatory subunit I	PPP2R2A	51.66	42.80	2.68	1	1	1
O75521	Enoyl-CoA delta isomerase 2, mitochondrial	ECI2	43.56	42.77	2.28	1	1	1
P51570	Galactokinase	GALK1	42.25	42.66	2.81	1	1	1
O95819	Iitogen-activated protein kinase kinase kinase kinase	MAP4K4	142.01	42.58	1.05	1	1	1
Q9UKS6	in kinase C and casein kinase substrate in neurons pro	PACSIN3	48.46	42.51	2.59	1	1	1
O14936	Peripheral plasma membrane protein CASK	CASK	105.06	42.33	1.08	1	1	1
Q14008	Cytoskeleton-associated protein 5	CKAP5	225.35	42.30	0.69	1	1	1
Q13835	Plakophilin-1	PKP1	82.81	42.29	0.8	1	1	1
P84103	Serine/arginine-rich splicing factor 3	SRSF3	19.32	42.25	8.54	1	1	1
P26038	Moesin	MSN	67.78	42.05	1.73	1	1	1
Q9NRZ9	Lymphoid-specific helicase	HELLS	97.01	42.04	1.31	1	1	1
P00492	Hypoxanthine-guanine phosphoribosyltransferase	HPRT1	24.56	41.83	5.96	1	1	1
P26639	Threonine--tRNA ligase, cytoplasmic	TARS	83.38	41.79	1.66	1	1	1
P11310	um-chain specific acyl-CoA dehydrogenase, mitochoı	ACADM	46.56	41.70	3.56	1	1	1
Q9UET6	e tRNA (cytidine(32)/guanosine(34)-2'-O)-methyltran	FTSJ1	36.06	41.36	3.95	1	1	1
Q9BZE4	Nucleolar GTP-binding protein 1	GTPBP4	73.92	41.13	1.58	1	1	1
Q9UNF1	Melanoma-associated antigen D2	MAGED2	64.91	40.72	1.98	1	1	1
Q15269	Periodic tryptophan protein 2 homolog	PWP2	102.39	40.71	1.2	1	1	1
Q3MHD2	Protein LSM12 homolog	LSM12	21.69	40.62	7.18	1	1	1
Q8NEE6	Dynein regulatory complex subunit 6	FBXL13	83.87	40.54	0.95	1	1	1
Q9BSD7	Cancer-related nucleoside-triphosphatase	NTPCR	20.70	40.31	7.89	1	1	1
Q9UBX3	Mitochondrial dicarboxylate carrier	SLC25A10	31.26	40.30	2.79	1	1	1
Q9UNQ2	Probable dimethyladenosine transferase	DIMT1	35.21	40.19	3.51	1	1	1
P35249	Replication factor C subunit 4	RFC4	39.66	39.96	3.58	1	1	1
P22531	Small proline-rich protein 2E	SPRR2E	7.85	39.89	25	1	1	1
O43837	citrate dehydrogenase [NAD] subunit beta, mitochond	IDH3B	42.16	39.82	4.16	1	1	1
P18077	60S ribosomal protein L35a	RPL35A	12.53	39.58	6.36	1	1	1
P48735	Isocitrate dehydrogenase [NADP], mitochondrial	IDH2	50.88	39.41	2.88	1	1	1
Q9NTJ3	Structural maintenance of chromosomes protein 4	SMC4	147.09	39.31	0.47	1	1	1
O60684	Importin subunit alpha-7	KPNA6	59.99	39.04	1.31	1	1	1
Q14690	Protein RRP5 homolog	PDCD11	208.57	38.64	0.53	1	1	1
Q96N67	Dedicator of cytokinesis protein 7	DOCK7	242.41	38.54	0.37	1	1	1
O43395	U4/U6 small nuclear ribonucleoprotein Prp3	PRPF3	77.48	36.65	1.02	1	1	1
Q9Y2R5	28S ribosomal protein S17, mitochondrial	MRPS17	14.49	36.20	6.92	1	1	1
P62333	26S proteasome regulatory subunit 10B	PSMC6	44.15	35.72	2.31	1	1	1
P06454	Prothymosin alpha	PTMA	12.20	35.30	12.61	1	1	1
P32926	Desmoglein-3	DSG3	107.47	34.80	0.7	1	1	1
P27695	DNA-(apurinic or apyrimidinic site) lyase	APEX1	35.53	34.53	4.72	1	1	1
Q9UHX1	Poly(U)-binding-splicing factor PUF60	PUF60	59.84	33.75	5.55	1	1	1
Q8IX12	Cell division cycle and apoptosis regulator protein 1	CCAR1	132.74	30.52	0.78	1	1	1
Q15019	Septin-2	SEPT2	41.46	29.22	2.49	1	1	1

Supplementary Table 2

REAGENT or RESOURCE	RESOURCE	IDENTIFIER
**Antibodies**		
Mouse anti-Vinculin	Santa Cruz Biotechnology	Cat# SC-73614 RRID: AB_1131294
Rabbit anti-PPARγ	Cell Signaling Technology	Cat# 2435 RRID:AB_2166051
Rabbit anti-FASN	Absin	Cat# abs133825
Rabbit anti-ACC	Cell Signaling Technology	Cat# 3676 RRID:AB_2219397
Rabbit anti-ACLY	Cell Signaling Technology	Cat# 9441 RRID:AB_331805
Rabbit anti-GAPDH	Cell Signaling Technology	Cat# 5174 RRID:AB_10622025
Rabbit anti-PPARα	Proteintech	Cat# 15540-1-AP RRID:AB_2252506
Rabbit anti-PPARδ	Proteintech	Cat# 10156-2-AP RRID:AB_2252532
Rabbit anti-SREBF1	Proteintech	Cat# 14088-1-AP RRID:AB_2255217
Mouse anti-FLAG Tag	Proteintech	Cat# 20543-1-AP RRID:AB_11232216
Mouse anti-MYC Tag	Proteintech	Cat# 60003-2-Ig RRID:AB_2734122
Mouse anti-HA Tag	Santa Cruz Biotechnology	Cat# SC-7392 RRID:AB_627809
Mouse anti-His Tag	Santa Cruz Biotechnology	Cat# SC-8036
Rabbit anti-V5 Tag	Proteintech	Cat#14440-1-AP
Rabbit anti-USP1	Proteintech	Cat# 14346-1-AP RRID:AB_2214314
Rabbit anti-USP3	Proteintech	Cat# 12490-1-AP RRID:AB_10639042
Rabbit anti-USP11	Proteintech	Cat# 10244-1-AP RRID:AB_2288400
Rabbit anti-USP13	Proteintech	Cat# 16840-1-AP RRID:AB_2214569
Rabbit anti-USP14	Proteintech	Cat# 14517-1-AP RRID:AB_2257124
Rabbit anti-USP15	Proteintech	Cat# 14354-1-AP RRID:AB_2257148
Rabbit anti-USP17	Abcam	Cat# ab174914
Rabbit anti-USP21	Proteintech	Cat# 17856-1-AP

Rabbit anti-USP22	Sigma-Aldrich	Cat# HPA044980<br>RRID:AB_10794503
Mouse anti-USP22	Santa Cruz Biotechnology	Cat# SC-390585
Rabbit anti-USP24	Proteintech	Cat# 13126-1-AP<br>RRID:AB_2212761
Rabbit anti-USP26	elabscience	Cat# EAP4323
Rabbit anti-USP27	Absin	Cat# abs102919
Mouse anti-USP29	Santa Cruz Biotechnology	Cat# sc-517145
Rabbit anti-USP33	Proteintech	Cat# 20445-1-AP<br>RRID:AB_10694439
Rabbit anti-USP34	Proteintech	Cat# 18827-1-AP<br>RRID:AB_2213348
Rabbit anti-USP36	Proteintech	Cat# 14783-1-AP<br>RRID:AB_2213357
Rabbit anti-USP37	Proteintech	Cat# 18465-1-AP<br>RRID:AB_10598483
Rabbit anti-USP39	Proteintech	Cat# 23865-1-AP
Rabbit anti-USP46	Proteintech	Cat# 13502-1-AP<br>RRID:AB_10642951
Rabbit anti-USP48	Proteintech	Cat# 12076-1-AP<br>RRID:AB_2213840
Rabbit anti-USP51	Abnova	Cat# H00158880-A01<br>RRID:AB_463546
Rabbit anti-USP54	Abcam	Cat# ab151001
Mouse anti-PPARγ	Santa Cruz Biotechnology	Cat# SC-7273
Rabbit anti-LaminB1	Cell Signaling Technology	Cat# 13435S
Rabbit anti-P-AKT (Ser 473)	Cell Signaling Technology	Cat# 4060S
Rabbit anti-CPT1A	Proteintech	Cat# 15184-1-AP
Rabbit anti-CPT2	Proteintech	Cat# 26555-1-AP
Rabbit anti-ACOX1	Proteintech	Cat# 10957-1-AP
Rabbit anti-ACADL	Proteintech	Cat# 17526-1-AP
Rabbit anti-ECHS1	Proteintech	Cat# 11305-1-AP
Rabbit anti-pVHL	Proteintech	Cat# 24756-1-AP
Rabbit anti-CUL4B	Proteintech	Cat# 12916-1-AP
Rabbit anti-SCD1	Abcam	Cat# ab236868

Mouse anti-Actin	Proteintech	Cat# 60008-1-Ig RRID:AB_2289225
**Chemicals, Peptides, and Recombinant Proteins**		
DMEM	Gibco	Cat# 11995065
RPMI 1640	Gibco	Cat# A1049101
RPMI 1640 no glucose	Gibco	Cat# 11879020
FBS	Gibco	Cat# 10099-141
13C-Glucose (U-13C6, 99%)	Cambridge Isotope Laboratories	Cat# CLM-1396;
EdU	Invitrogen	Cat# C10418
CCK-8 kit	MedChem Express	Cat# HY-K0301
MG 132	Selleckchem	Cat# S2619
CHX	Cell Signaling Technology	Cat# 2112S
S-Protein	Millipore, USA	Cat# 69704
IHC Kit	Proteintech, Wuhan, China	Cat# KIHC-5
PPARγ Transcription Factor Assay Kit	Abcam	Cat# ab133101
Nuclear Extraction Kit	Abcam	Cat# ab113474
Triglyceride Quantification Assay Kit	Solarbio	Cat# BC0625
Oil-Red-O staining	Solarbio	Cat# G1262
Pierce Agarose ChIP Kit	Thermo Fisher Scientific	Cat# 26156
AgeI	Thermo Fisher Scientific	Cat# FD1464
EcoRI	Thermo Fisher Scientific	Cat# FD0274
Blasticidin	InvivoGen	Cat# ant-bl-1
Puromycin	InvivoGen	Cat# ant-pr-1
Goat anti-mouse Alexa 488	Invitrogen	Cat# bs-0296G-AF488
Goat anti-rabbit Alexa 555	Invitrogen	Cat# bs-0295G-AF555
BCA protein assay kit	TIANGEN BIOTECH	Cat# PA115
Lipofectamine 2000	Invitrogen	Cat# 11668019
RNeasy Mini Kit	Qiagen	Cat# 74104
**Biological Samples**		
Human HCC Tissue	Shanghai Outdo Biotech	HLivH180Su11

**Microarrays**	Company	
Human HCC Tissue Microarrays	Shanxi ChaoYing Biotechnology Company	Lv1021
10 paired human HCC Tissues	The First Affiliated hospital of Dalian Medical University	N/A
**Deposited Data**		
HCC expression data	TCGA	www.cbioportal.org
**Experimental Models: Organisms/Strains**		
BALB/C nude mice	Beijing Vital River Laboratory Animal Technology Co., Ltd (Beijing, China)	N/A
**Recombinant DNA**		
pLKO.1-puro-shRNA		
USP22-shRNA-1 CCGGAGCTACC AGGAGTCCACA AAGCTCGAGCT TTGTGGACTCCT GGTAGCTTTTTG	Sigma-Aldrich	TRCN0000296867
USP22-shRNA-2 CCGGTGTGCCAGG ACTACATCTATGCTC GAGCATAGATGTAG TCCTGGCACATTTTTG	Sigma-Aldrich	TRCN0000296868
ACC-shRNA CCGGTACAAGGGATA CAGGTATTTACTCGA GTAAATACCTGTATC CCTTGTATTTTTG	Sigma-Aldrich	TRCN0000232456
ACLY-shRNA CCGGCGTGAGAGCA ATTCGAGATTACTCG AGTAATCTCGAATTG CTCTCACGTTTTTG	Sigma-Aldrich	TRCN0000291817
PPAR-shRNA CCGGCAGCATTTCT	Sigma-Aldrich	TRCN0000001673

ACTCCACATTACTCG<br>AGTAATGTGGAGTAG<br>AAATGCTGTTTTT		
SFB-PPARγ	This paper	N/A
Myc-USP22	This paper	N/A
Myc-USP22C185S	This paper	N/A
pLoc-USP22	This paper	N/A
Lenti- PPARγ	This paper	N/A
HA-Ub	Addgene	#17608
HA-Ub-K48	Addgene	#17605
HA-Ub- K63	Addgene	#17606
**Cell lines**		
MHCC-97H	CTCC, Shanghai, China	
HUH7	CTCC, Shanghai, China	SCSP-528
Bel-7402	CTCC, Shanghai, China	TCHu182/ SCSP-526
Hep3B	CTCC, Shanghai, China	TCHu 10
HepG2	CTCC, Shanghai, China	SCSP-5045
SMMC-7721	CTCC, Shanghai, China	SCSP-510
HEK293T	CTCC, Shanghai, China	TCHu 52
HCC-LM3	CTCC, Shanghai, China	GNHu44
PLC/PRF/5	CTCC, Shanghai, China	TCHu119
MHCC-97L	Liver Cancer Institute, Fudan University (China)	N/A
HB611	Liver Cancer Institute, Fudan University (China)	N/A
THLE-2	ATCC	CRL-2706
SNU449	ATCC	CRL-2234
**Software and Algorithms**		
Image J	N/A	https://imagej.nih.gov/ij/
Gene Set Enrichment Analysis (GSEA)	Broad Institute	http://software.broadinstitute.org/gsea/index.jsp
SPSS 21.0	IBM	N/A
GEPIA	N/A	http://gepia.cancer-pku.cn/
CCLE	N/A	https://portals.broadinstitute.org/ccle
METABOANALYST	N/A	https://www.metaboanalyst.ca/

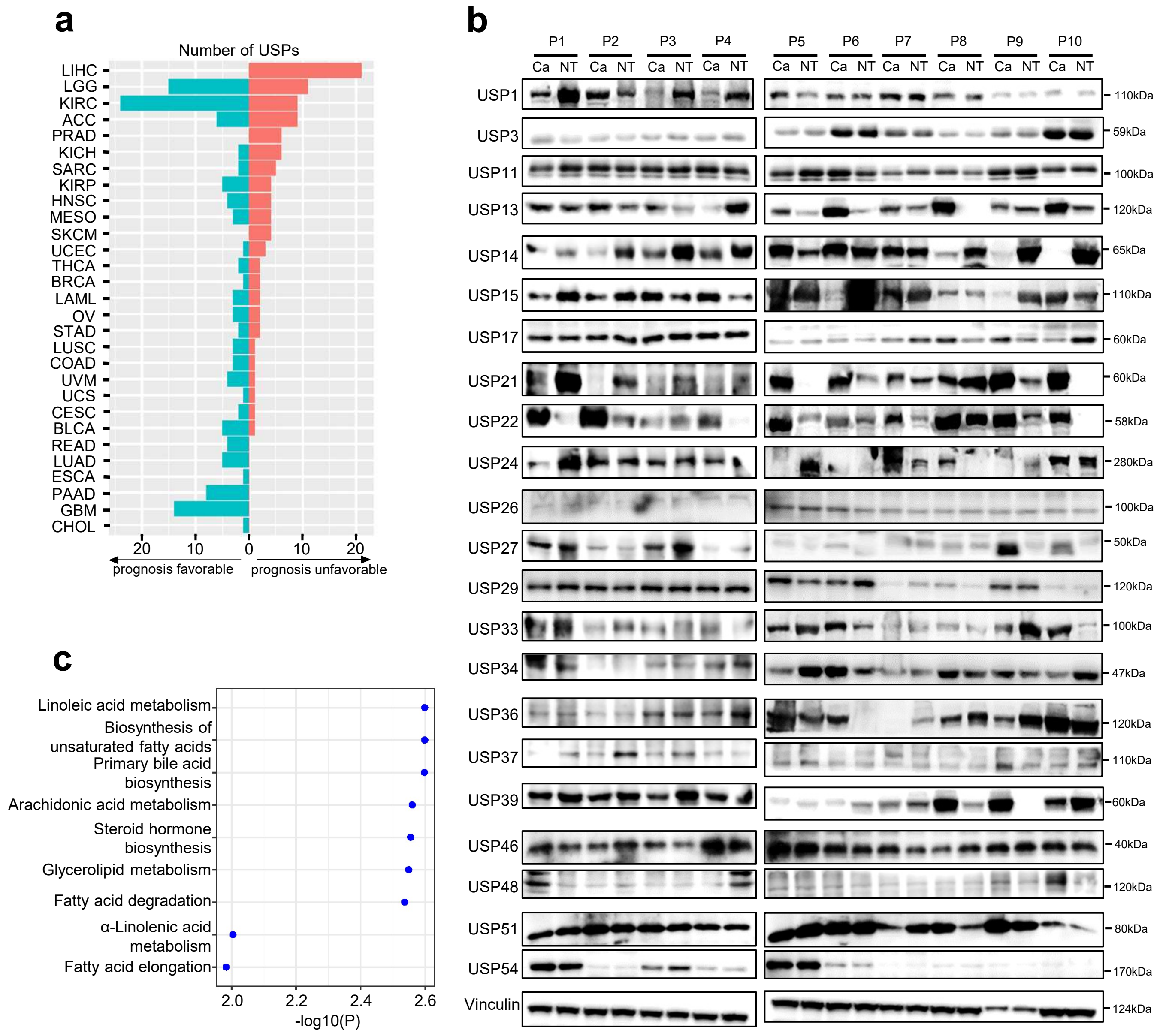

a
Number of USPs
LIHC
LGG
KIRC
ACC
PRAD
KICH
SARC
KIRP
HNSC
MESO
SKCM
UCEC
THCA
BRCA
LAML
OV
STAD
LUSC
COAD
UVM
UCS
CESC
BLCA
READ
LUAD
ESCA
PAAD
GBM
CHOL
20 10 0 10 20
prognosis favorable
prognosis unfavorable
b
P1 P2 P3 P4 P5 P6 P7 P8 P9 P10
Ca NT
USP1 110kDa
USP3 59kDa
USP11 100kDa
USP13 120kDa
USP14 65kDa
USP15 110kDa
USP17 60kDa
USP21 60kDa
USP22 58kDa
USP24 280kDa
USP26 100kDa
USP27 50kDa
USP29 120kDa
USP33 100kDa
USP34 47kDa
USP36 120kDa
USP37 110kDa
USP39 60kDa
USP46 40kDa
USP48 120kDa
USP51 80kDa
USP54 170kDa
Vinculin 124kDa
c
Linoleic acid metabolism
Biosynthesis of unsaturated fatty acids
Primary bile acid biosynthesis
Arachidonic acid metabolism
Steroid hormone biosynthesis
Glycerolipid metabolism
Fatty acid degradation
α-Linolenic acid metabolism
Fatty acid elongation
2.0 2.2 2.4 2.6
-log10(P)

Supplementary Figure 1 | Abnormal lipid metabolism in human HCC with high USP22 expression.

a, The number of USP family members associated with prognosis (red represents unfavorable, green represents favorable) in various tumors was analyzed by Kaplan–Meier analysis based on TCGA database. (LIHC: Liver hepatocellular carcinoma, LGG: Brain Lower Grade Glioma, KIRC: Kidney renal clear cell carcinoma, ACC: Adrenocortical carcinoma, PRAD: Prostate adenocarcinoma, KICH: Kidney Chromophobe, SARC: Sarcoma, KIRP: Kidney renal papillary cell carcinoma, HNSC: Head and Neck squamous cell carcinoma, MESO: Mesothelioma, SKCM: Skin Cutaneous Melanoma, UCEC: Uterine Corpus Endometrial Carcinoma, THCA: Thyroid carcinoma, BRCA: Breast invasive carcinoma, LAML: Acute Myeloid Leukemia, OV: Ovarian serous cystadenocarcinoma, STAD: Stomach adenocarcinoma, LUSC: Lung squamous cell carcinoma, COAD: Colon adenocarcinoma, UVM: Uveal Melanoma, UCS: Uterine Carcinosarcoma, CESC: Cervical squamous cell carcinoma and endocervical adenocarcinoma, BLCA: Bladder Urothelial Carcinoma, READ: Rectum adenocarcinoma, LUAD: Lung adenocarcinoma, ESCA: Esophageal carcinoma, PAAD: Pancreatic adenocarcinoma, GBM: Glioblastoma multiforme, CHOL: Cholangio carcinoma)

b, Western blot analysis of protein expression of USP family members (which associated with prognosis in HCC based on TCGA HCC database) in the 10 paired HCC cancer tissues and normal adjacent tissues.

c, Gene set enrichment analysis (GSEA) of metabolic pathways with USP22-correlated genes based on TCGA HCC database.

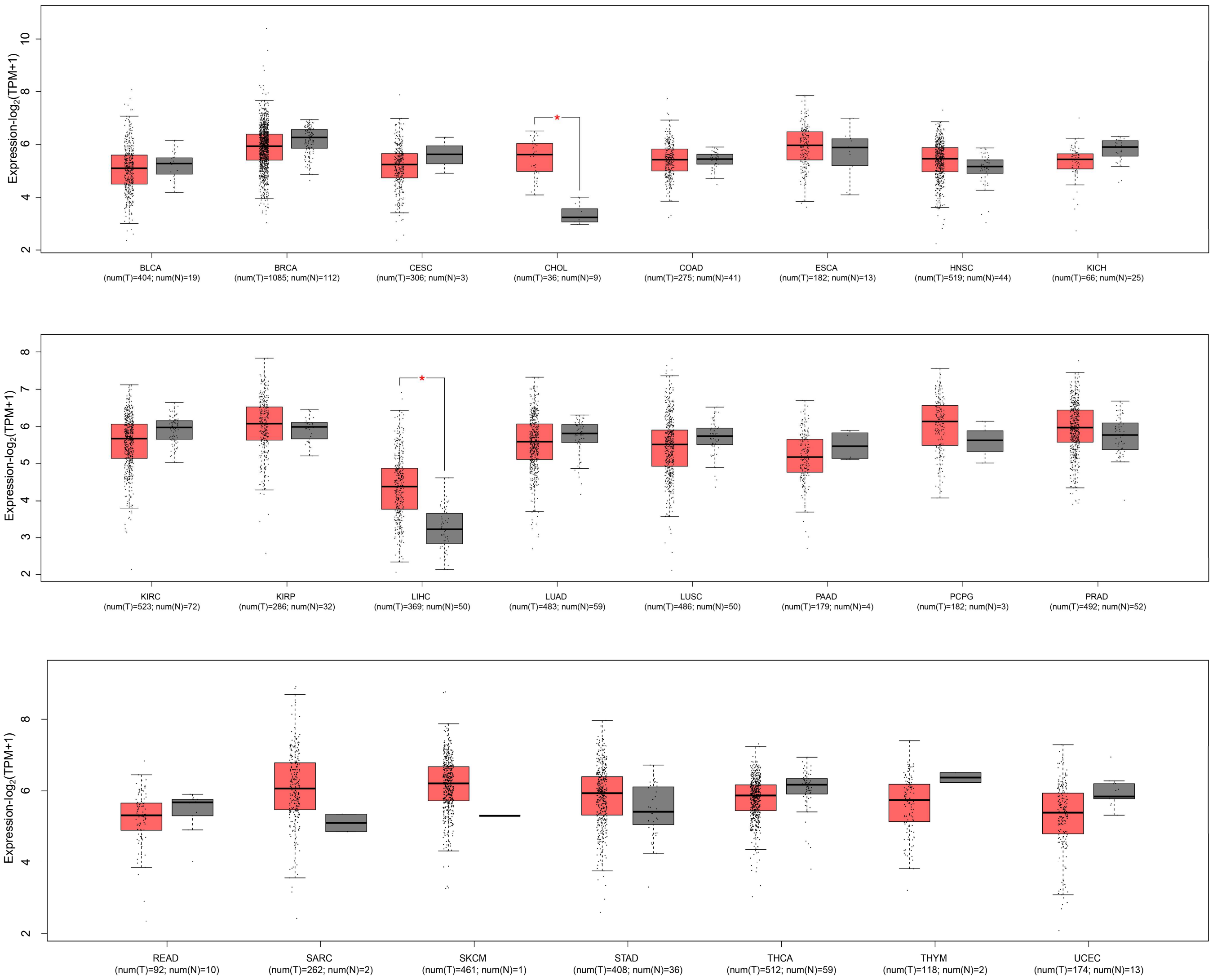

Supplementary Figure 2 | *USP22* is specifically highly expressed in HCC and CHOL cancers.

USP22 expression in various cancers based on TCGA database. The expression data is first $\log_2$ (TPM+1) transformed for differential analysis and the $\log_2$ FC is defined as median (Tumor) - median (Normal). T: tumor; N: normal tissue . Sample numbers (num) are shown below the corresponding box plot. One-way ANOVA test. (This data is exported from the website: http://gepia2.cancer-pku.cn/#index).

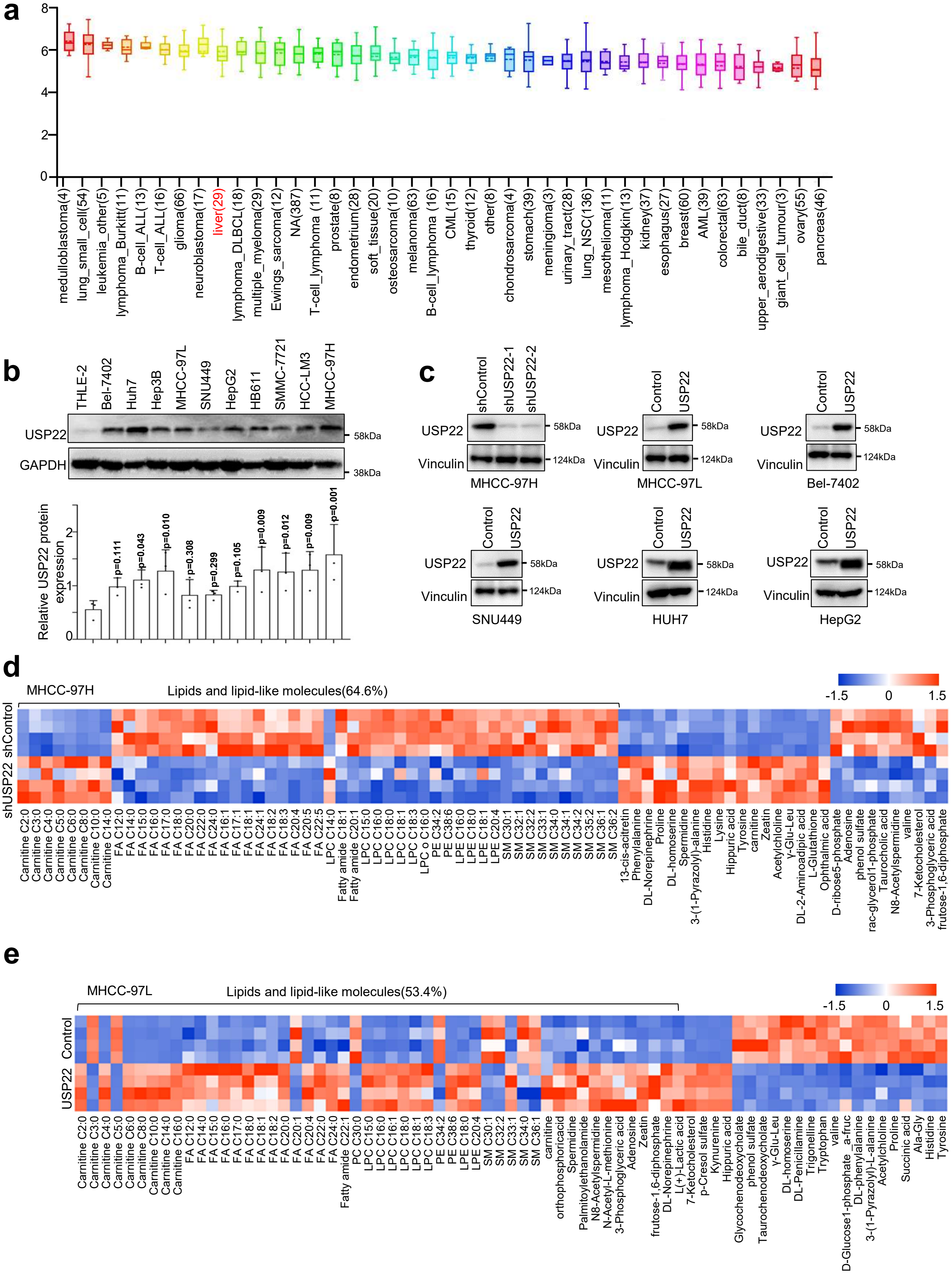
a
0
2
4
6
8
medulloblastoma(4)
lung_small_cell(54)
leukemia_other(5)
lymphoma_Burkitt(11)
B-cell_ALL(13)
T-cell_ALL(16)
glioma(66)
neuroblastoma(17)
liver(29)
lymphoma_DLBCL(18)
multiple_myeloma(29)
Ewings_sarcoma(12)
NA(387)
T-cell_lymphoma (11)
prostate(8)
endometrium(28)
soft_tissue(20)
osteosarcoma(10)
melanoma(63)
B-cell_lymphoma (16)
CML(15)
thyroid(12)
other(8)
chondrosarcoma(4)
stomach(39)
meningioma(3)
urinary_tract(28)
lung_NSC(136)
mesothelioma(11)
lymphoma_Hodgkin(13)
kidney(37)
esophagus(27)
breast(60)
AML(39)
colorectal(63)
bile_duct(8)
upper_aerodigestive(33)
giant_cell_tumour(3)
ovary(55)
pancreas(46)
b
THLE-2
Bel-7402
Huh7
Hep3B
MHCC-97L
SNU449
HepG2
HB611
SMMC-7721
HCC-LM3
MHCC-97H
USP22
58kDa
GAPDH
38kDa
Relative USP22 protein expression
0
1
2
p=0.111
p=0.043
p=0.010
p=0.308
p=0.299
p=0.105
p=0.009
p=0.012
p=0.009
p=0.001
c
shControl
shUSP22-1
shUSP22-2
USP22
58kDa
Vinculin
124kDa
MHCC-97H
Control
USP22
USP22
58kDa
Vinculin
124kDa
MHCC-97L
Control
USP22
USP22
58kDa
Vinculin
124kDa
Bel-7402
Control
USP22
USP22
58kDa
Vinculin
124kDa
SNU449
Control
USP22
USP22
58kDa
Vinculin
124kDa
HUH7
Control
USP22
USP22
58kDa
Vinculin
124kDa
HepG2
d
MHCC-97H
Lipids and lipid-like molecules(64.6%)
-1.5
0
1.5
shControl
shUSP22
Carnitine C2:0
Carnitine C3:0
Carnitine C4:0
Carnitine C5:0
Carnitine C6:0
Carnitine C10:0
Carnitine C14:0
FA C12:0
FA C14:0
FA C15:0
FA C16:0
FA C17:0
FA C18:0
FA C20:0
FA C22:0
FA C24:0
FA C16:1
FA C17:1
FA C18:1
FA C24:1
FA C18:2
FA C18:3
FA C20:4
FA C20:5
FA C22:5
LPC C14:0
Fatty amide C18:1
Fatty amide C20:1
LPC C15:0
LPC C16:0
LPC C18:0
LPC C16:1
LPC C18:1
LPC C18:3
LPC o C16:0
PE C34:2
PE C38:6
LPE C16:0
LPE C18:0
LPE C18:1
LPE C20:4
SM C30:1
SM C32:1
SM C32:2
SM C33:1
SM C34:0
SM C34:1
SM C34:2
SM C35:2
SM C36:1
SM C36:2
13-cis-acitretin
Phenylalanine
DL-Norepinephrine
Proline
DL-homoserine
Spermidine
3-(1-Pyrazolyl)-alanine
Histidine
Lysine
Hippuric acid
Tyrosine
carnitine
Zeatin
Acetylcholine
γ-Glu-Leu
DL-2-Aminoadipic acid
L-Glutathione
Ophthalmic acid
D-ribose5-phosphate
Adenosine
phenol sulfate
rac-glycerol1-phosphate
Taurocholic acid
N8-Acetylspermidine
valine
7-Ketocholesterol
3-Phosphoglyceric acid
frutose-1,6-diphosphate
e
MHCC-97L
Lipids and lipid-like molecules(53.4%)
-1.5
0
1.5
Control
USP22
Carnitine C2:0
Carnitine C3:0
Carnitine C4:0
Carnitine C5:0
Carnitine C6:0
Carnitine C8:0
Carnitine C10:0
Carnitine C14:0
Carnitine C16:0
FA C12:0
FA C14:0
FA C15:0
FA C16:0
FA C17:0
FA C18:0
FA C18:1
FA C18:2
FA C20:0
FA C20:1
FA C20:4
FA C22:0
FA C24:0
Fatty amide C22:1
PC C30:0
LPC C15:0
LPC C16:0
LPC C16:1
LPC C18:0
LPC C18:1
LPC C18:3
PE C34:2
PE C38:6
LPE C18:0
LPE C20:4
SM C30:1
SM C32:2
SM C33:1
SM C34:0
SM C36:1
carnitine
orthophosphoricacid
Spermidine
Palmitoylethanolamide
N8-Acetylspermidine
N-Acetyl-L-methionine
3-Phosphoglyceric acid
Adenosine
Zeatin
frutose-1,6-diphosphate
DL-Norepinephrine
L(+)-Lactic acid
7-Ketocholesterol
p-Cresol sulfate
Kynurenine
Hippuric acid
Glycochenodeoxycholate
phenol sulfate
Taurochenodeoxycholate
γ-Glu-Leu
DL-homoserine
DL-Penicillamine
Trigonelline
Tryptophan
valine
D-Glucose1-phosphate_a-fruc
DL-phenylalanine
3-(1-Pyrazolyl)-L-alanine
Acetylcholine
Proline
Succinic acid
Ala-Gly
Histidine
Tyrosine

Supplementary Figure 3 | USP22 is highly expressed in hepatocellular carcinoma cells and promotes lipids and lipid-like molecules accumulation.

a, *USP22* expression was analyzed among 1,457 human cancer cell lines in CCLE database (This data is exported from the website: https://portals.broadinstitute.org/ccle. The numbers indicate different cell lines in each cancer type).

b, Western blot analysis of USP22 expression in 10 HCC cell lines (Bel-7402, HUH7, Hep3B, MHCC-97L, SUN-449, HepG2, HB611, SMMC-7721, HCC-LM3 and MHCC-97H) and 1 liver cell line (THLE-2). Bottom panel is quantification of USP22 protein levels. Data are presented as mean values ± SD. One-way ANOVA test. n=3 independent experiments.

c, Construction of USP22-knockdown (MHCC-97H-shUSP22-1/2) and overexpression (MHCC-97L-USP22, Bel-7402-USP22, SNU449-USP22, HUH7-USP22 and HepG2-USP22) stable HCC cell lines. USP22 proteins were analyzed in stable cells by western blot.

d, Heatmap analysis of significantly changed metabolites (n=79) in MHCC-97H-shUSP22-1 cells. LC-MS–based nontargeted metabolomic analysis, and the data were standardized by total peak area. $p < 0.05$, unpaired two-tailed Student's t test. Red indicates increase, and blue indicates decrease. -1.5~1.5 indicates the Fold Change.

e, Heatmap analysis of significantly changed metabolites (n=73) in MHCC-97L-USP22 cells. LC-MS–based nontargeted metabolomic analysis, and the data were standardized by total peak area. $p < 0.05$, unpaired two-tailed Student's t test. Red indicates increase, and blue indicates decrease. -1.5~1.5 indicates the Fold Change. Source data are provided in the Source Data file.

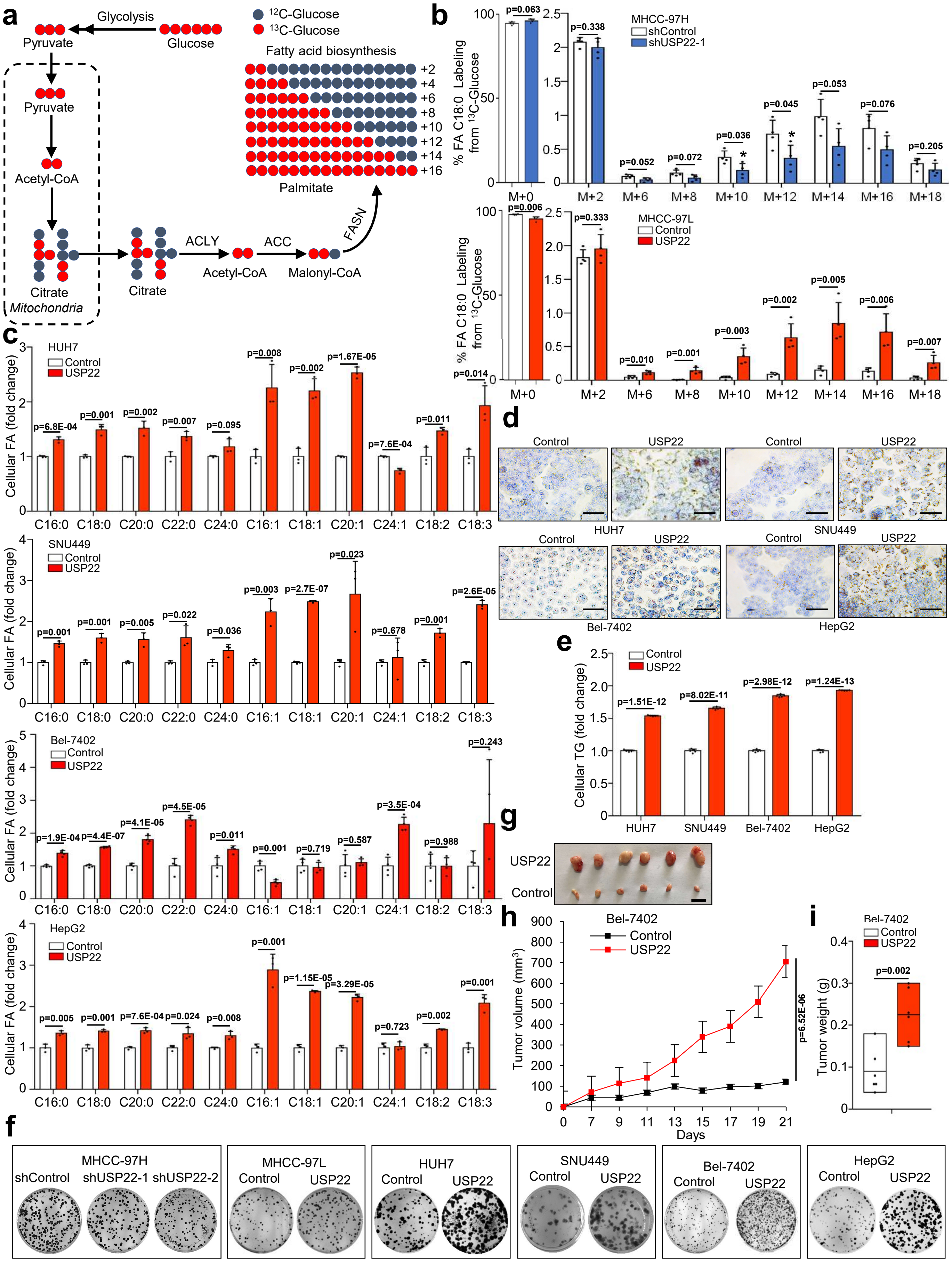

a
Glycolysis
Pyruvate
Glucose
12C-Glucose
13C-Glucose
Fatty acid biosynthesis
+2
+4
+6
+8
+10
+12
+14
+16
Palmitate
Acetyl-CoA
Citrate
Mitochondria
ACLY
ACC
FASN
Malonyl-CoA
b
% FA C18:0 Labeling from 13C-Glucose
MHCC-97H
shControl
shUSP22-1
p=0.063
p=0.338
p=0.052
p=0.072
p=0.036
p=0.045
p=0.053
p=0.076
p=0.205
M+0
M+2
M+6
M+8
M+10
M+12
M+14
M+16
M+18
MHCC-97L
Control
USP22
p=0.006
p=0.333
p=0.010
p=0.001
p=0.003
p=0.002
p=0.005
p=0.006
p=0.007
c
Cellular FA (fold change)
HUH7
Control
USP22
p=6.8E-04
p=0.001
p=0.002
p=0.007
p=0.095
p=0.008
p=0.002
p=1.67E-05
p=7.6E-04
p=0.011
p=0.014
C16:0
C18:0
C20:0
C22:0
C24:0
C16:1
C18:1
C20:1
C24:1
C18:2
C18:3
SNU449
p=0.001
p=0.001
p=0.005
p=0.022
p=0.036
p=0.003
p=2.7E-07
p=0.023
p=0.678
p=0.001
p=2.6E-05
Bel-7402
p=1.9E-04
p=4.4E-07
p=4.1E-05
p=4.5E-05
p=0.011
p=0.001
p=0.719
p=0.587
p=3.5E-04
p=0.988
p=0.243
HepG2
p=0.005
p=0.001
p=7.6E-04
p=0.024
p=0.008
p=0.001
p=1.15E-05
p=3.29E-05
p=0.723
p=0.002
p=0.001
d
Control
USP22
HUH7
SNU449
Bel-7402
HepG2
e
Cellular TG (fold change)
p=1.51E-12
p=8.02E-11
p=2.98E-12
p=1.24E-13
HUH7
SNU449
Bel-7402
HepG2
g
USP22
Control
h
Bel-7402
Tumor volume (mm3)
Days
p=6.52E-06
i
Tumor weight (g)
p=0.002
f
MHCC-97H
shControl
shUSP22-1
shUSP22-2
MHCC-97L
HUH7
SNU449
Bel-7402
HepG2

Supplementary Figure 4 | USP22 promotes *de novo* synthesis of fatty acids and tumorigenesis of HCC.

a, Diagram of de novo synthesis of fatty acids from ^{13}C-glucose.

b, The percentages of various isotopomers of FA 18:0 after trace to [U-^{13}C] glucose in MHCC-97H-shUSP22-1 and MHCC-97L-USP22 cells. Medium was changed to RPMI 1640 containing [U-^{13}C]-glucose (2 g/L) when the cell density was about 80%, and 24 hours later cell culture plates were washed with PBS and snap-frozen in liquid nitrogen and subjected to LC-MS analysis. n=4 independent biological experiments.

c, The relative content of FAs (C16:0, C18:0, C20:0, C22:0, C24:0, C16:1, C18:1, C20:1, C24:1, C18:2 and C18:3) was analyzed in HUH7-USP22, SNU449-USP22, HepG2-USP22 and Bel-7402-USP22 cells by LC-MS–based nontargeted metabolomic analysis, and the data were standardized by total peak area. The experiments were repeated triplicates (for HUH7, HepG2 and SNU449) or four times (Bel-7402).

d, Oil red staining assay in in HUH7-USP22, SNU449-USP22, HepG2-USP22 and Bel-7402 cells. Cells were analyzed 24 hours after plating. Scale bars, 50 μm.

e, The relative content of TG was assayed in HUH7-USP22, SNU449-USP22, HepG2-USP22 and Bel-7402 cells. Cells were analyzed 24 hours after plating. The data shown represent the means (±SD) of biological triplicates. n=5 biologically independent samples.

f, Clone formation assay in MHCC-97H-shUSP22-1, MHCC-97L-USP22, HUH7-USP22, Bel-7402-USP22, SNU449-USP22 and HepG2-USP22 cells.

g, h and i, Bel-7402-USP22 or control cells were injected into the right flank of male nude mice. Tumor volumes were measured every 3 days. Tumor images (g), growth curves (h) and weight (i) were obtained at day 21 after dissection. n=6 biologically independent tumor samples. Scale bars, 1 cm. Data in b, c, e and h are presented as mean values ± SD and data in i are presented as mean values with minima and maxima. Unpaired two-tailed Student's t test. ns, nonsignificant. Source data are provided in the Source Data file.

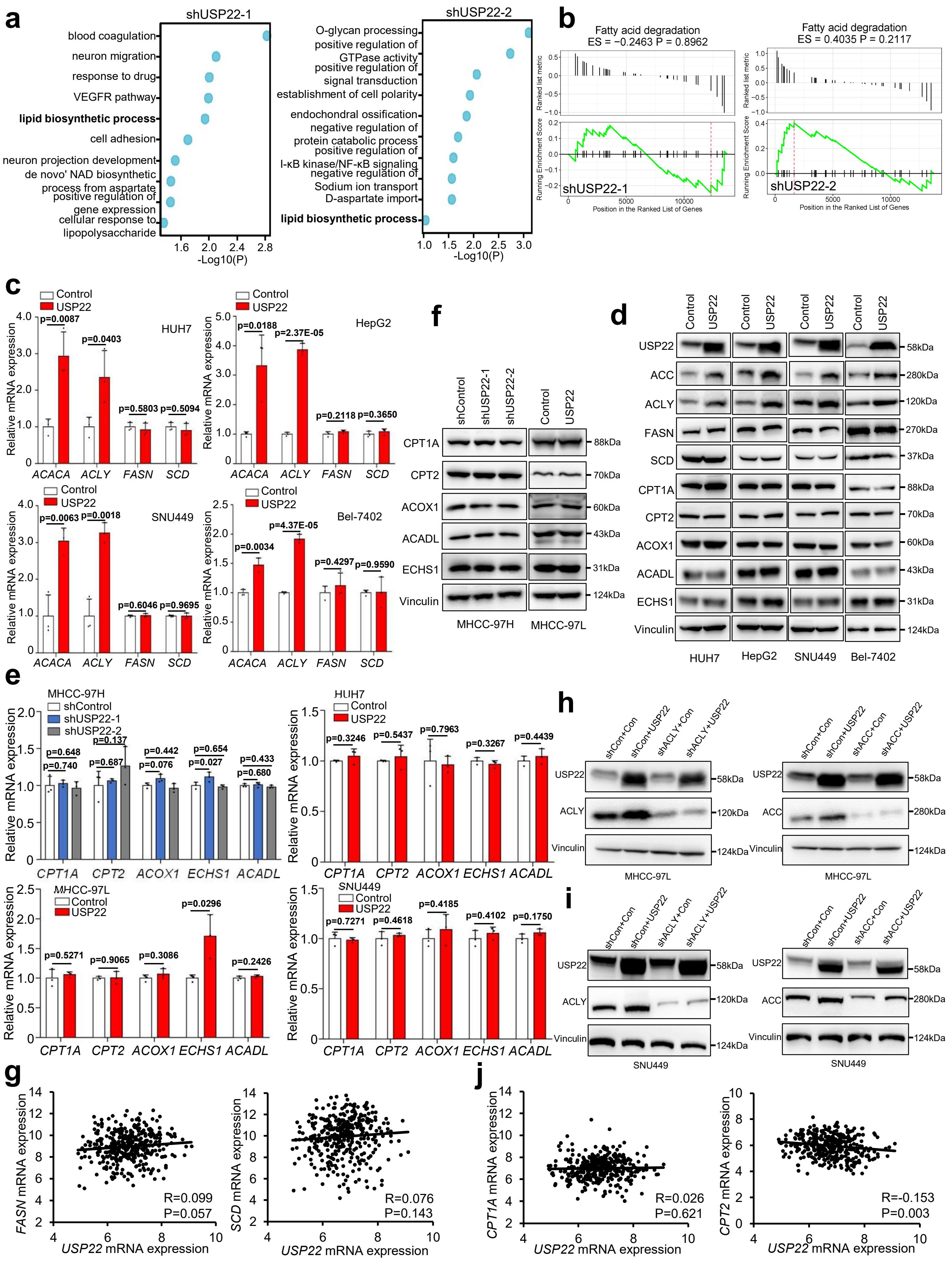

a
shUSP22-1
blood coagulation
neuron migration
response to drug
VEGFR pathway
lipid biosynthetic process
cell adhesion
neuron projection development
de novo' NAD biosynthetic process from aspartate
positive regulation of gene expression
cellular response to lipopolysaccharide
-Log10(P)
shUSP22-2
O-glycan processing
positive regulation of GTPase activity
positive regulation of signal transduction
establishment of cell polarity
endochondral ossification
negative regulation of protein catabolic process
positive regulation of I-κB kinase/NF-κB signaling
negative regulation of Sodium ion transport
D-aspartate import
lipid biosynthetic process
-Log10(P)
b
Fatty acid degradation
ES = −0.2463 P = 0.8962
shUSP22-1
Fatty acid degradation
ES = 0.4035 P = 0.2117
shUSP22-2
Ranked list metric
Running Enrichment Score
Position in the Ranked List of Genes
c
Control
USP22
HUH7
HepG2
SNU449
Bel-7402
Relative mRNA expression
ACACA ACLY FASN SCD
p=0.0087 p=0.0403 p=0.5803 p=0.5094
p=0.0188 p=2.37E-05 p=0.2118 p=0.3650
p=0.0063 p=0.0018 p=0.6046 p=0.9695
p=0.0034 p=4.37E-05 p=0.4297 p=0.9590
f
shControl shUSP22-1 shUSP22-2 Control USP22
CPT1A CPT2 ACOX1 ACADL ECHS1 Vinculin
88kDa 70kDa 60kDa 43kDa 31kDa 124kDa
MHCC-97H MHCC-97L
d
Control USP22
USP22 ACC ACLY FASN SCD CPT1A CPT2 ACOX1 ACADL ECHS1 Vinculin
58kDa 280kDa 120kDa 270kDa 37kDa 88kDa 70kDa 60kDa 43kDa 31kDa 124kDa
HUH7 HepG2 SNU449 Bel-7402
e
MHCC-97H
shControl
shUSP22-1
shUSP22-2
p=0.648 p=0.740 p=0.687 p=0.137 p=0.442 p=0.076 p=0.654 p=0.027 p=0.433 p=0.680
HUH7
Control
USP22
p=0.3246 p=0.5437 p=0.7963 p=0.3267 p=0.4439
MHCC-97L
Control
USP22
p=0.5271 p=0.9065 p=0.3086 p=0.0296 p=0.2426
SNU449
Control
USP22
p=0.7271 p=0.4618 p=0.4185 p=0.4102 p=0.1750
Relative mRNA expression
CPT1A CPT2 ACOX1 ECHS1 ACADL
h
shCon+Con shCon+USP22 shACLY+Con shACLY+USP22
USP22 ACLY Vinculin
58kDa 120kDa 124kDa
MHCC-97L
shCon+Con shCon+USP22 shACC+Con shACC+USP22
USP22 ACC Vinculin
58kDa 280kDa 124kDa
MHCC-97L
i
shCon+Con shCon+USP22 shACLY+Con shACLY+USP22
USP22 ACLY Vinculin
58kDa 120kDa 124kDa
SNU449
shCon+Con shCon+USP22 shACC+Con shACC+USP22
USP22 ACC Vinculin
58kDa 280kDa 124kDa
SNU449
g
FASN mRNA expression
R=0.099
P=0.057
SCD mRNA expression
R=0.076
P=0.143
USP22 mRNA expression
j
CPT1A mRNA expression
R=0.026
P=0.621
CPT2 mRNA expression
R=-0.153
P=0.003
USP22 mRNA expression

Supplementary Figure 5 | USP22 upregulates ACC and ACLY expression.

a, GO-BP (biological process) analysis was performed using significantly different genes ($Log_2FC > 1$, $Q < 0.05$) in the transcriptome data of MHCC-97H-shUSP22-1/2 cells. (https://david.ncifcrf.gov/.)

b, Gene set enrichment analysis of fatty acid degradation gene sets in the expression profiles of MHCC-97H cells transduced with two independent USP22 shRNAs.

c, qRT-PCR analysis of the key metabolic enzymes of *de novo* fatty acid synthesis (*ACC*, *ACLY*, *FASN* and *SCD1*) in HUH7-USP22, HepG2-USP22, SNU449-USP22 and Bel-7402-USP22 cells. Unpaired two-tailed Student's t test. The data shown represent the means (±SD) of three biological replicates.

d, Western blot analysis of metabolic enzymes of *de novo* fatty acid synthesis (ACC, ACLY, FASN and SCD1) and fatty acid degradation (CPT1A, CPT2, ACOX1, ACADL and ECHS1) in HUH7-USP22, HepG2-USP22, SNU449-USP22 and Bel-7402-USP22 cells.

e, qRT-PCR analysis of metabolic enzymes of fatty acid degradation (*CPT1A*, *CPT2*, *ACOX1*, *ACADL* and *ECHS1*) in MHCC-97H-sh1/2, MHCC-97L-USP22, HUH7-USP22 and SNU449-USP22 cells. One-way ANOVA test. The data shown represent the means (±SD) of three biological replicates.

f, Western blot analysis of metabolic enzymes of fatty acid degradation (CPT1A, CPT2, ACOX1, ACADL and ECHS1) in MHCC-97H-sh1/2 and MHCC-97L-USP22 cells.

g, Correlation analysis between *USP22* and *FASN*, *SCD* based on the TCGA HCC database. R represents the pearson correlation coefficient.

h and i, Transduced USP22 into ACC knockdown MHCC-97L and or ACLY knockdown SNU449 cells. Immunoblotted with USP22, ACLY, ACC and Vinculin antibodies.

j, Correlation analysis between *USP22* and *CPT1A*, *CPT2* based on the TCGA HCC database. R represents the Pearson correlation coefficient. Source data are provided in the Source Data file.

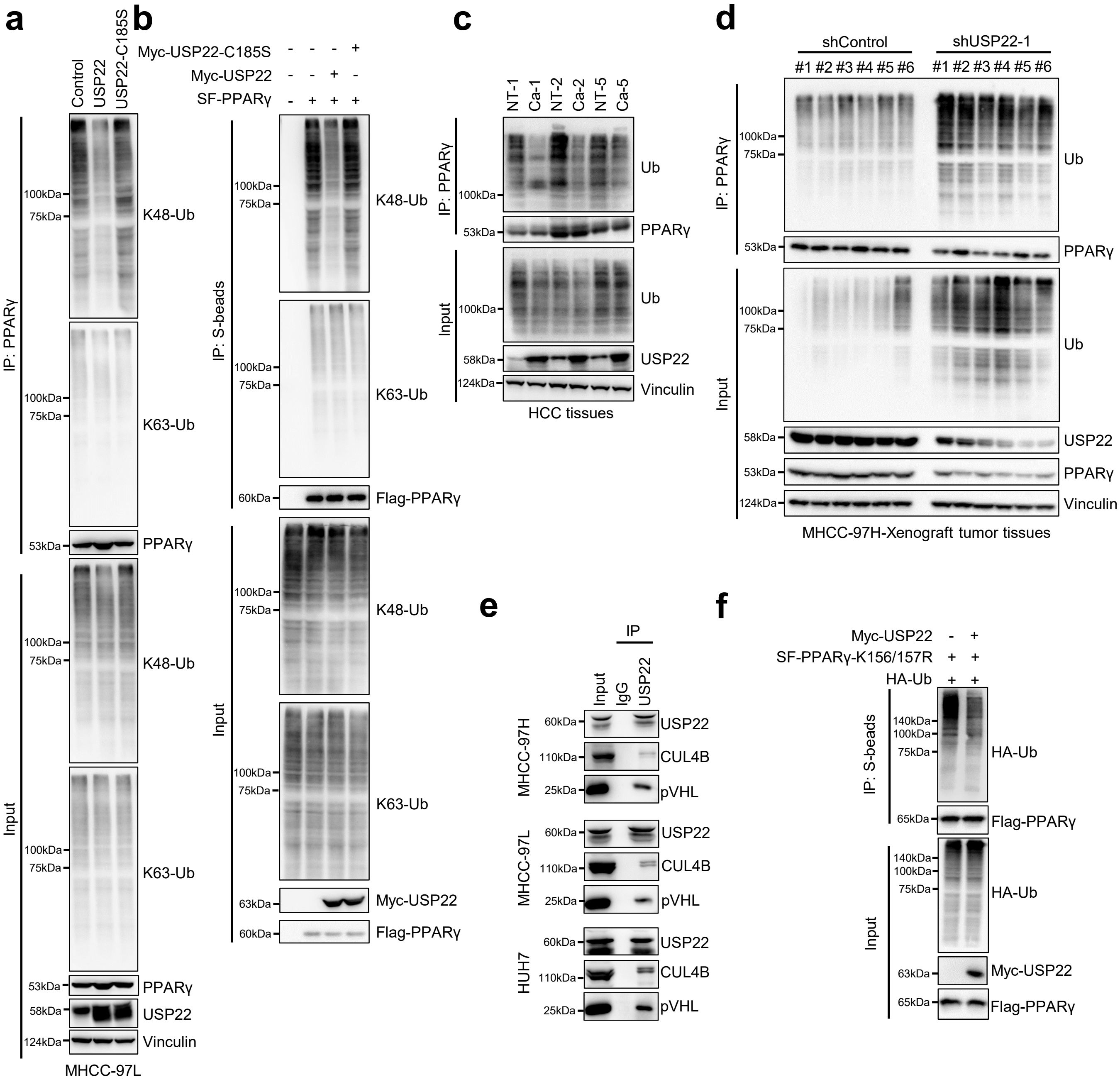

a
Control
USP22
USP22-C185S
IP: PPARγ
K48-Ub
K63-Ub
PPARγ
Input
K48-Ub
K63-Ub
PPARγ
USP22
Vinculin
MHCC-97L
b
Myc-USP22-C185S
Myc-USP22
SF-PPARγ
IP: S-beads
K48-Ub
K63-Ub
Flag-PPARγ
Input
K48-Ub
K63-Ub
Myc-USP22
Flag-PPARγ
c
NT-1
Ca-1
NT-2
Ca-2
NT-5
Ca-5
IP: PPARγ
Ub
PPARγ
Input
Ub
USP22
Vinculin
HCC tissues
d
shControl
shUSP22-1
#1 #2 #3 #4 #5 #6 #1 #2 #3 #4 #5 #6
IP: PPARγ
Ub
PPARγ
Input
Ub
USP22
PPARγ
Vinculin
MHCC-97H-Xenograft tumor tissues
e
IP
Input
IgG
USP22
MHCC-97H
MHCC-97L
HUH7
USP22
CUL4B
pVHL
f
Myc-USP22
SF-PPARγ-K156/157R
HA-Ub
IP: S-beads
HA-Ub
Flag-PPARγ
Input
HA-Ub
Myc-USP22
Flag-PPARγ

Supplementary Figure 6 | USP22 deubiquitinates PPARγ.

a, Ubiquitination assay of PPARγ in MHCC-97L-USP22, MHCC-97L-USP22 C185S cells treated with 10 μM MG132 for 6 hours. The specific K48 and K63-Ub antibodies were used to detect the ubiquitination of PPARγ.

b, Ubiquitination assay of PPARγ in HEK293T cells cotransfected with Myc-USP22, Myc-USP22-C185S and Flag-PPARγ and treated with 10 μM MG132 for 6 hours. The Specific K48 and K63-Ub antibodies were used to detect the ubiquitination of PPARγ.

c, Ubiquitination assay of PPARγ in HCC cancer tissues and adjacent normal tissues (Patient #1, #2 and #5).

d, Ubiquitination assay of PPARγ in tumors derived from MHCC-97H-shUSP22-1 and shControl cells.

e, Cell lysates of MHCC-97H, MHCC-97L and HUH7 were immunoprecipitated with IgG or USP22 antibodies, and immunoblotted with antibodies against USP22, $CRL4B^{AhR}$ and pVHL.

f, Ubiquitination assay of PPARγ-K156/157R in HEK293T cells cotransfected with HA-Ub, SF-PPARγ-K156/157R, Myc-USP22 and treated with 10 μM MG132 for 6 hours.

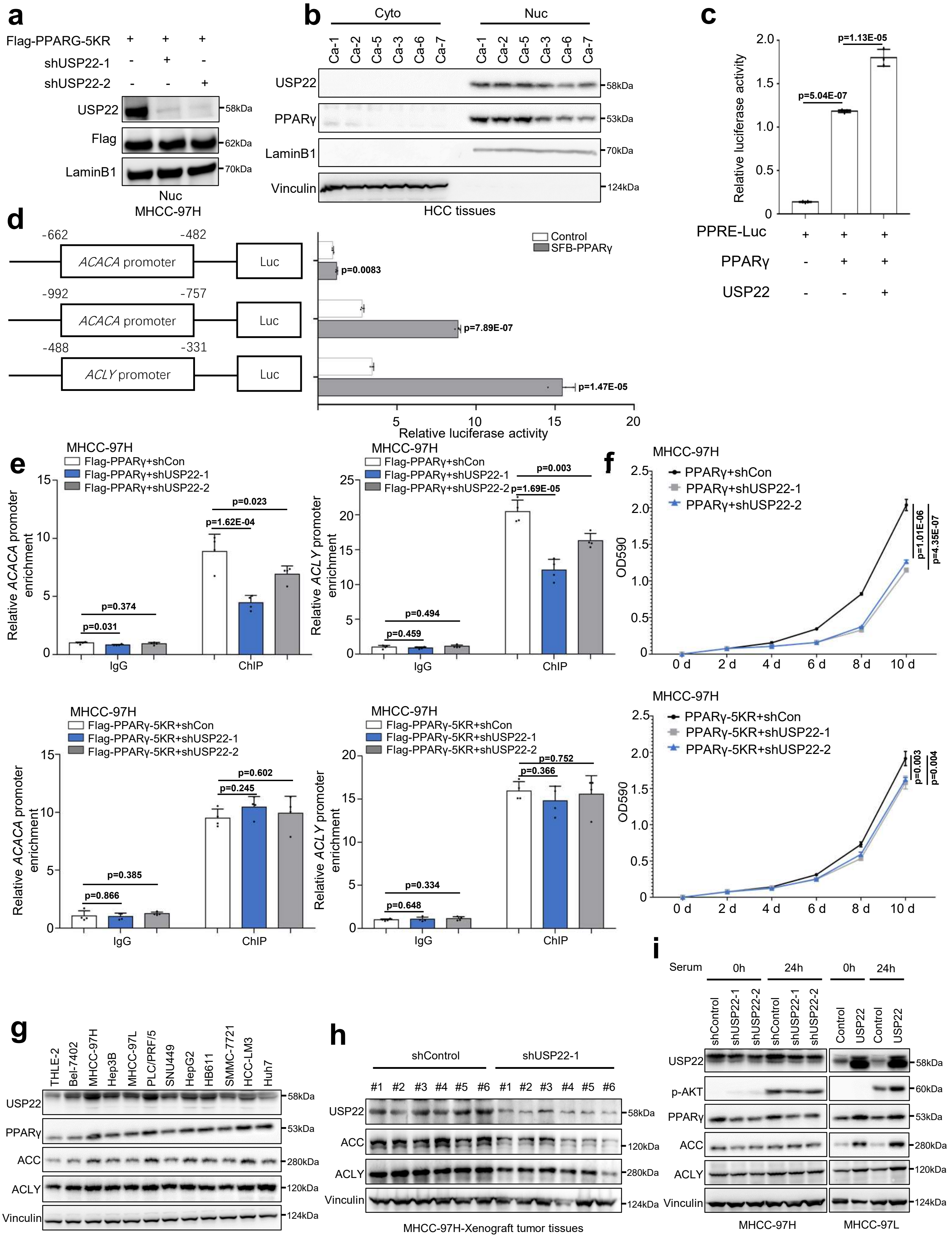
a
Flag-PPARG-5KR + + +
shUSP22-1 - + -
shUSP22-2 - - +
USP22
Flag
LaminB1
58kDa
62kDa
70kDa
Nuc
MHCC-97H
b
Cyto
Nuc
Ca-1 Ca-2 Ca-5 Ca-3 Ca-6 Ca-7
USP22
PPARγ
LaminB1
Vinculin
58kDa
53kDa
70kDa
124kDa
HCC tissues
c
Relative luciferase activity
p=5.04E-07
p=1.13E-05
PPRE-Luc + + +
PPARγ - + +
USP22 - - +
d
-662 -482
ACACA promoter
Luc
-992 -757
ACACA promoter
Luc
-488 -331
ACLY promoter
Luc
Control
SFB-PPARγ
p=0.0083
p=7.89E-07
p=1.47E-05
Relative luciferase activity
e
MHCC-97H
Flag-PPARγ+shCon
Flag-PPARγ+shUSP22-1
Flag-PPARγ+shUSP22-2
Relative ACACA promoter enrichment
p=0.374
p=0.031
p=0.023
p=1.62E-04
IgG
ChIP
Relative ACLY promoter enrichment
p=0.494
p=0.459
p=0.003
p=1.69E-05
Flag-PPARγ-5KR+shCon
Flag-PPARγ-5KR+shUSP22-1
Flag-PPARγ-5KR+shUSP22-2
p=0.385
p=0.866
p=0.602
p=0.245
p=0.334
p=0.648
p=0.752
p=0.366
f
MHCC-97H
PPARγ+shCon
PPARγ+shUSP22-1
PPARγ+shUSP22-2
OD590
p=1.01E-06
p=4.35E-07
0 d 2 d 4 d 6 d 8 d 10 d
PPARγ-5KR+shCon
PPARγ-5KR+shUSP22-1
PPARγ-5KR+shUSP22-2
p=0.003
p=0.004
g
THLE-2 Bel-7402 MHCC-97H Hep3B MHCC-97L PLC/PRF/5 SNU449 HepG2 HB611 SMMC-7721 HCC-LM3 Huh7
USP22
PPARγ
ACC
ACLY
Vinculin
58kDa
53kDa
280kDa
120kDa
124kDa
h
shControl
shUSP22-1
#1 #2 #3 #4 #5 #6
USP22
ACC
ACLY
Vinculin
58kDa
120kDa
280kDa
124kDa
MHCC-97H-Xenograft tumor tissues
i
Serum
0h
24h
shControl shUSP22-1 shUSP22-2
Control USP22
USP22
p-AKT
PPARγ
ACC
ACLY
Vinculin
58kDa
60kDa
53kDa
280kDa
120kDa
124kDa
MHCC-97H
MHCC-97L

Supplementary Figure 7 | USP22 expression correlated with PPARγ, ACC and ACLY.

a, Western blot analysis of Flag-PPARγ-5KR expression in nucleus fractions of MHCC-97H- Flag-PPARγ-5KR and MHCC-97H-Flag-PPARγ-5KR-shUSP22 cells.

b, Western blot analysis of USP22 and PPARγ expression in cytoplasmic and nucleus fractions of HCC cancer tissues. Patients #1, #2 and #5, USP22 high expression. Patient #3, #6, #7, USP22 low expression.

c, HEK293T cells were transfected with indicated plasmids. Cells were harvested after 24 hours transfected and the luciferase activity was measured by Promega Dual-Luciferase Reporter assay system. Data are presented as mean values ± SD. One-way ANOVA test. n=3 biologically independent samples.

d, *ACACA*/*ACLY* promoter plasmid was transfected into HEK293T cells with/without SFB-PPARγ. Cells were harvested after 24 hours transfected and promoter activity was measured by Promega Dual-Luciferase Reporter assay system. The data shown represent the means (±SD) of three biological replicates. Unpaired two-tailed Student's t test.

e, ChIP analysis of PPARγ or PPARγ-5KR binding to the *ACLY* and *ACACA* promoters in MHCC-97H-PPARγ+shUSP22 cells and MHCC-97H-PPARγ-5KR +shUSP22 cells. qPCR was performed with specific primers to the PPARγ-binding motifs. Data were normalized to the input and presented as mean values ± SD. ANOVA test. n=3 biologically independent experiments.

f, Proliferation assays were performed on MHCC-97H- PPARγ+shUSP22cells and MHCC-97H- PPARγ-5KR+shUSP22 cells. Data are presented as mean values ± SD. One-way ANOVA test. n=3 biologically independent samples.

g, Western blot analysis of USP22, PPARγ, ACC and ACLY protein expression in 11 HCC cell lines (Bel-7402, MHCC-97H, Hep3B, MHCC-97L, PLC/PRF/5, SUN-449, HepG2, HB611, SMMC-7721, HCC-LM3 and HUH7) and one normal liver cell line (THLE-2).

h, Western blot analysis of USP22, ACC and ACLY expression in tumors derived from MHCC-97H-shUSP22-1 and shControl cells.

i, Western blot analysis of p-AKT(Ser473), USP22, PPARγ, ACC and ACLY expression in MHCC-97H-shUSP22 and USP22 transduced MHCC-97L cells, which deprived serum for 6 hours then incubated with or without 10% serum media for 24 hours. Source data are provided in the Source Data file.

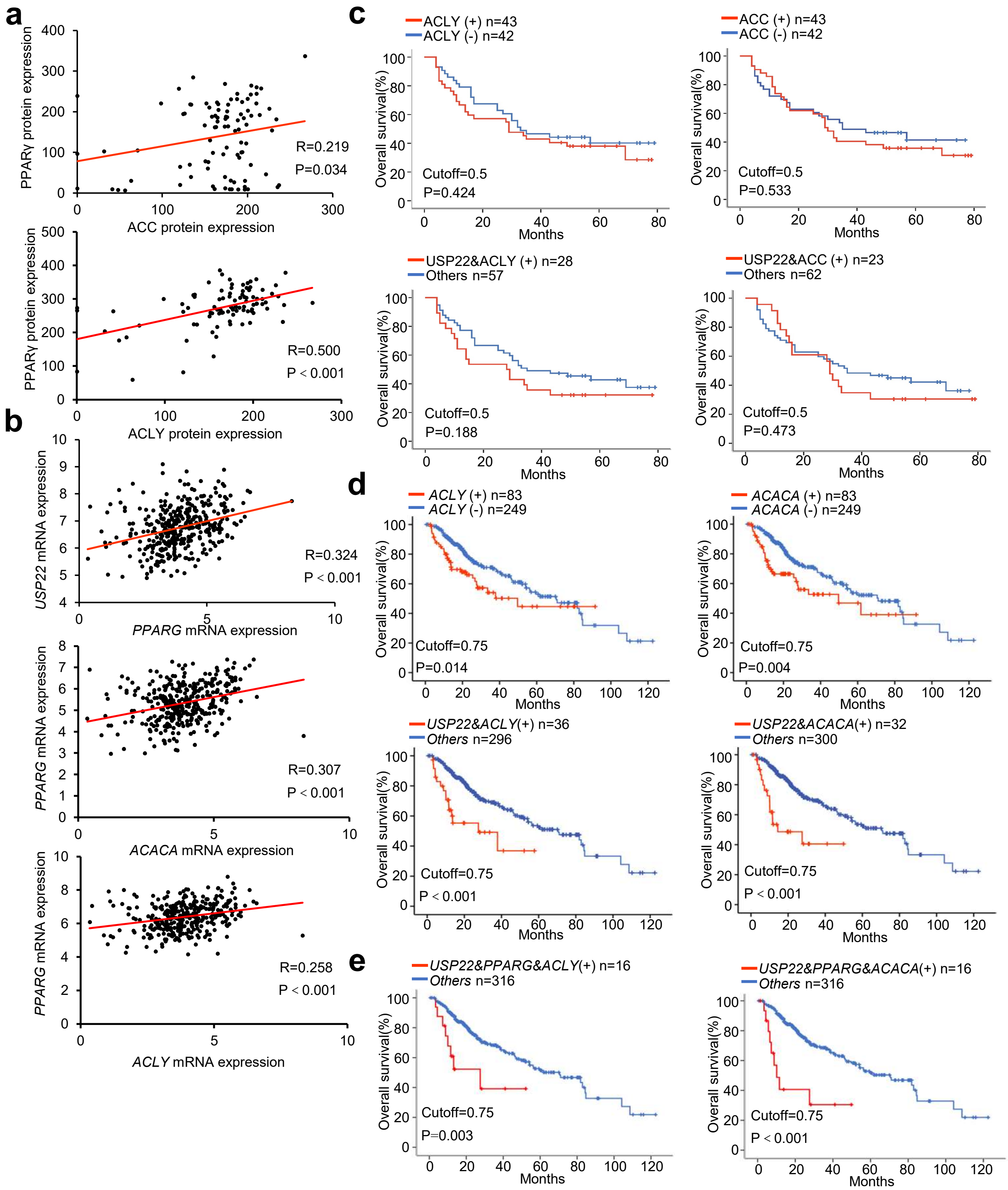
a
PPARγ protein expression
ACC protein expression
R=0.219
P=0.034
PPARγ protein expression
ACLY protein expression
R=0.500
P < 0.001
b
USP22 mRNA expression
PPARG mRNA expression
R=0.324
P < 0.001
PPARG mRNA expression
ACACA mRNA expression
R=0.307
P < 0.001
PPARG mRNA expression
ACLY mRNA expression
R=0.258
P < 0.001
c
ACLY (+) n=43
ACLY (-) n=42
Overall survival(%)
Cutoff=0.5
P=0.424
Months
ACC (+) n=43
ACC (-) n=42
Cutoff=0.5
P=0.533
USP22&ACLY (+) n=28
Others n=57
Cutoff=0.5
P=0.188
USP22&ACC (+) n=23
Others n=62
Cutoff=0.5
P=0.473
d
ACLY (+) n=83
ACLY (-) n=249
Cutoff=0.75
P=0.014
ACACA (+) n=83
ACACA (-) n=249
Cutoff=0.75
P=0.004
USP22&ACLY(+) n=36
Others n=296
Cutoff=0.75
P < 0.001
USP22&ACACA(+) n=32
Others n=300
Cutoff=0.75
P < 0.001
e
USP22&PPARG&ACLY(+) n=16
Others n=316
Cutoff=0.75
P=0.003
USP22&PPARG&ACACA(+) n=16
Others n=316
Cutoff=0.75
P < 0.001

Supplementary Figure 8 | The USP22–PPARγ/ACC/ACLY axis contributed to HCC prognosis.

a, Correlation analysis between PPARγ and ACC, PPARγ and ACLY protein expression based on H-Score in HCC TMAs (HLivH180Su11). R represents the Pearson correlation coefficient.

b, Correlation analysis between *USP22* and *PPARG*, *PPARG* and *ACACA*, *PPARG* and *ACLY* based on TCGA HCC database. R represents Pearson correlation coefficient.

c, Kaplan–Meier curves of the survival analysis of ACC-positive, ACLY-positive, USP22&ACLY copositive and USP22&ACC co-positive patients based on HCC Tissue Microarray prognosis data (HLivH180Su11).

d, Kaplan–Meier curves of survival analysis of *ACLY*-positive, ACC-positive, *USP22&ACLY* co-positive and *USP22&ACACA* copositive patients based on HCC TCGA database. Cutoff = 0.75.

e, Kaplan–Meier curves of survival analysis of *USP22&PPARG&ACLY* co-positive and *USP22&PPARG&ACACA* copositive patients based on HCC TCGA database. Cutoff = 0.75. Source data are provided in the Source Data file.
