# Supplementary material for: USP22 regulates lipidome accumulation by stabilizing PPARγ in hepatocellular carcinoma
Source: Nat Commun. 2022 Apr 21;13:2187. doi: 10.1038/s41467-022-29846-9 (PMC9023467; doi:10.1038/s41467-022-29846-9)
Supplement: Supplementary file 3 — Reporting Summary [file 41467_2022_29846_MOESM3_ESM.pdf]

nature portfolio

Corresponding author(s): Hai-long Piao
Last updated by author(s): Feb 25, 2022

Reporting Summary

Nature Portfolio wishes to improve the reproducibility of the work that we publish. This form provides structure for consistency and transparency in reporting. For further information on Nature Portfolio policies, see our Editorial Policies and the Editorial Policy Checklist.

Statistics

For all statistical analyses, confirm that the following items are present in the figure legend, table legend, main text, or Methods section.

n/a	Confirmed	
☐	☒	The exact sample size (n) for each experimental group/condition, given as a discrete number and unit of measurement
☐	☒	A statement on whether measurements were taken from distinct samples or whether the same sample was measured repeatedly
☐	☒	The statistical test(s) used AND whether they are one- or two-sided<br>*Only common tests should be described solely by name; describe more complex techniques in the Methods section.*
☒	☐	A description of all covariates tested
☒	☐	A description of any assumptions or corrections, such as tests of normality and adjustment for multiple comparisons
☐	☒	A full description of the statistical parameters including central tendency (e.g. means) or other basic estimates (e.g. regression coefficient) AND variation (e.g. standard deviation) or associated estimates of uncertainty (e.g. confidence intervals)
☐	☒	For null hypothesis testing, the test statistic (e.g. F, t, r) with confidence intervals, effect sizes, degrees of freedom and P value noted<br>*Give P values as exact values whenever suitable.*
☒	☐	For Bayesian analysis, information on the choice of priors and Markov chain Monte Carlo settings
☒	☐	For hierarchical and complex designs, identification of the appropriate level for tests and full reporting of outcomes
☐	☒	Estimates of effect sizes (e.g. Cohen's d, Pearson's r), indicating how they were calculated

Our web collection on statistics for biologists contains articles on many of the points above.

Software and code

Policy information about availability of computer code

Data collection	Thermo Scientific Xcalibur (Ver. 4.2.47, Thermo Fisher Scientific, USA) was used for raw data collection of metabolic and lipid profiling. Thermo Trace Finder EFS (Ver. 3.2.512.0, Thermo Fisher Scientific, USA) was used for data processing, integrating peak area and deriving Excel table. Thermo Scientific Proteome Discoverer(Ver.1.3, Thermo Fisher Scientific, USA) was used for processing of proteomic data.
Data analysis	All of the relative protein expression was normalized by ImageJ (version no.: 1.8.0_112; https://imagej.nih.gov/ij/). Statistical analysis was performed using the SPSS 21.0 software package (SPSS, Inc., Chicago, IL, USA).

For manuscripts utilizing custom algorithms or software that are central to the research but not yet described in published literature, software must be made available to editors and reviewers. We strongly encourage code deposition in a community repository (e.g. GitHub). See the Nature Portfolio guidelines for submitting code & software for further information.

Data

Policy information about availability of data

All manuscripts must include a data availability statement. This statement should provide the following information, where applicable:

- Accession codes, unique identifiers, or web links for publicly available datasets
- A description of any restrictions on data availability
- For clinical datasets or third party data, please ensure that the statement adheres to our policy

All the experiment data that support the findings of this study are included within the paper, its Supplementary Information files, Source Data files and public repositories and also available from the corresponding author upon reasonable request. The raw RNA-seq data used in this study are available in the Sequence Read Archive (SRA, https://www.ncbi.nlm.nih.gov/sra/) under the Bioproject accession PRJNA809499. The LC-MS/MS data for PPARγ interactome are available within the article and its Supplementary Table 2. The data used in this study for gene expression profiling interactive analysis are available in The Cancer Genome Atlas (TCGA;

https://tcga-data.nci.nih.gov/). Promoter sequences are available from Eukaryotic Promoter Database (EPD; https://epd.epfl.ch/). The reference library used in RNA-seq analysis (Mus musculus GRCm38.dna.primary_assembly.fa) is available in the GeneRIF (https://www.ncbi.nlm.nih.gov/gene/?term=GRCm38). The source data underlying Fig 1, 2, 3, 5, 6, 7 and 8 as well as Supplementary Fig 3, 4, 5, 7 and 8 are provided as a Source Data file. All the other data supporting the findings of this study are available within the article and its Supplementary Information files. Source data are provided with this paper.

Field-specific reporting

Please select the one below that is the best fit for your research. If you are not sure, read the appropriate sections before making your selection.

- [x] Life sciences
- [ ] Behavioural & social sciences
- [ ] Ecological, evolutionary & environmental sciences

For a reference copy of the document with all sections, see nature.com/documents/nr-reporting-summary-flat.pdf

Life sciences study design

All studies must disclose on these points even when the disclosure is negative.

Sample size

Sample sizes for each experiment are provided in figure legends. For in vitro experiments, three biological replicates were achieved. Such sample sizes are typical for the in vitro experiments and sufficient for a statistical analysis. For in vivo assay, n=6 or n=8 mice were used.
No statistical method was used to determine the sample size. The sample sizes are determined empirically, and are similar in size to most existing studies in the field.

Data exclusions

No data were excluded from the analysis.

Replication

Each in vitro experiment was replicated at least triple successfully. At least six mice and human samples were used in this study and 3 replicates were successfully carried out for each sample.

Randomization

For animal studies, the mice were earmarked before grouping and then were randomly separated into groups by an independent person. And also other in vitro experiments, such as cells/samples were randomly assigned to examination groups.

Blinding

Experimenters were blinded to group allocation for IHC staining and grading, as well as quantification of CFU numbers. The research staff who performed the RNA-sequencing library preparation had no knowledge about the sample characteristics. The investigators were not blinded to sample allocation during experiment and outcome assessment, because results used were obtained using objective quantitative methods.

Reporting for specific materials, systems and methods

We require information from authors about some types of materials, experimental systems and methods used in many studies. Here, indicate whether each material, system or method listed is relevant to your study. If you are not sure if a list item applies to your research, read the appropriate section before selecting a response.

Materials & experimental systems

n/a	Involved in the study	
[ ]	[x]	Antibodies
[ ]	[x]	Eukaryotic cell lines
[x]	[ ]	Palaeontology and archaeology
[ ]	[x]	Animals and other organisms
[ ]	[x]	Human research participants
[x]	[ ]	Clinical data
[x]	[ ]	Dual use research of concern

Methods

n/a	Involved in the study	
[x]	[ ]	ChIP-seq
[x]	[ ]	Flow cytometry
[x]	[ ]	MRI-based neuroimaging

Antibodies

Antibodies used

Mouse anti-Vinculin (1:1000) Santa Cruz Biotechnology Cat# SC-73614 RRID: AB_1131294
Rabbit anti-LaminB1 (1:1000) Cell Signaling Technology Cat# 13435S
Rabbit anti-PPARγ (1:1000) Cell Signaling Technology Cat# 2435 RRID:AB_2166051
Mouse anti-PPAR (1:500) Santa Cruz Biotechnology Cat# SC-7273
Rabbit anti-FASN (1:1000) Absin Cat# abs133825
Rabbit anti-ACC (1:1000) Cell Signaling Technology Cat# 3676S RRID:AB_2219397
Rabbit anti-ACLY (1:1000) Cell Signaling Technology Cat# 9441S RRID:AB_331805
Rabbit anti-GAPDH (1:3000) Cell Signaling Technology Cat# 5174S RRID:AB_10622025
Rabbit anti-PPARα (1:1000) Proteintech Cat# 15540-1-AP RRID:AB_2252506
Rabbit anti-PPARδ (1:1000) Proteintech Cat# 10156-2-AP RRID:AB_2252532
Rabbit anti-SREBF1(1:1000) Proteintech Cat# 14088-1-AP RRID:AB_2255217
Mouse anti-FLAG Tag (1:1000) Proteintech Cat# 20543-1-AP RRID:AB_11232216

Mouse anti-MYC Tag (1:1000) Proteintech Cat# 60003-2-Ig RRID:AB_2734122
Mouse anti-HA Tag (1:2000) Santa Cruz Biotechnology Cat# SC-7392 RRID:AB_627809
Mouse anti-His Tag (1:2000) Santa Cruz Biotechnology Cat# SC-8036
Rabbit anti-V5 Tag (1:1000) Proteintech Cat#14440-l-AP
Rabbit anti-USP1 (1:1000) Proteintech Cat# 14346-1-AP RRID:AB_2214314
Rabbit anti-USP3 (1:1000) Proteintech Cat# 12490-1-AP RRID:AB_10639042
Rabbit anti-USP11 (1:1000) Proteintech Cat# 10244-1-AP RRID:AB_2288400
Rabbit anti-USP13 (1:1000) Proteintech Cat# 16840-1-AP RRID:AB_2214569
Rabbit anti-USP14 (1:1000) Proteintech Cat# 14517-1-AP RRID:AB_2257124
Rabbit anti-USP15 (1:1000) Proteintech Cat# 14354-1-AP RRID:AB_2257148
Rabbit anti-USP17 (1:1000) Abcam Cat# ab174914
Rabbit anti-USP21 (1:1000) Proteintech Cat# 17856-1-AP
Rabbit anti-USP22 (1:1000) Sigma-Aldrich Cat# HPA044980 RRID:AB_10794503
Mouse anti-USP22 (1:500) Santa Cruz Biotechnology sc-390585
Rabbit anti-USP24 (1:1000) Proteintech Cat# 13126-1-AP RRID:AB_2212761
Rabbit anti-USP26 (1:1000) elabscience Cat# EAP4323
Rabbit anti-USP27 (1:1000) Absin Cat# abs102919
Mouse anti-USP29 (1:200) Santa Cruz Biotechnology Cat# sc-517145
Rabbit anti-USP33 (1:1000) Proteintech Cat# 20445-1-AP RRID:AB_10694439
Rabbit anti-USP34 (1:1000) Proteintech Cat# 18827-1-AP RRID:AB_2213348
Rabbit anti-USP36 (1:1000) Proteintech Cat# 14783-1-AP RRID:AB_2213357
Rabbit anti-USP37 (1:1000) Proteintech Cat# 18465-1-AP RRID:AB_10598483
Rabbit anti-USP39 (1:1000) Proteintech Cat# 23865-1-AP
Rabbit anti-USP46 (1:1000) Proteintech Cat# 13502-1-AP RRID:AB_10642951
Rabbit anti-USP48 (1:1000) Proteintech Cat# 12076-1-AP RRID:AB_2213840
Rabbit anti-USP51 (1:1000) Abnova Cat# H00158880-A01 RRID:AB_463546
Rabbit anti-USP54 (1:1000) Abcam Cat# ab151001
Rabbit anti-SCD1 (1:1000) Abcam Cat# ab236868
Rabbit anti-p-AKT(Ser 473) (1:1000) Cell Signaling Technology Cat# 4060S
Rabbit anti-CPT1A (1:1000) Proteintech Cat# 15184-1-AP
Rabbit anti-CPT2 (1:1000) Proteintech Cat# 26555-1-AP
Rabbit anti-ACOX1 (1:1000) Proteintech Cat# 10957-1-AP
Rabbit anti-ACADL (1:1000) Proteintech Cat# 17526-1-AP
Rabbit anti-ECHS1 (1:1000) Proteintech Cat# 11305-1-AP
Rabbit anti-pVHL (1:1000) Proteintech Cat# 24756-1-AP
Rabbit anti-CUL4B (1:1000) Proteintech Cat# 12916-1-AP
Mouse anti-Actin (1:2000) Proteintech Cat# 60008-1-Ig RRID:AB_2289225

Validation

Mouse anti-Vinculin, Human and Mouse, WB IP IF IHC, (https://www.scbt.com/zh/p/vinculin-antibody-7f9?requestFrom=search); In this paper for WB..
Rabbit anti-LaminB1, WB IP, (https://www.cellslgnal.cn/products/primary-antibodies/lamin-bl-d9v6h-rabbit-mab/134352site-searchtype=Products&N=4294956287&Ntt=13435s&fromPage=plp&_requestid=26798); In this paper for WB.
Rabbit anti-PPARγ, Human and Mouse, WB CHIP IF IHC, (https://www.cellsignal.co.uk/products/primary-antibodies/pparg-c26h12-rabbit-mab/2435?N=0+4294956287&Nrpp=200&No=4400&fromPage=plp); In this paper for WB CHIP IF and IHC.
Mouse anti-PPARγ, WB IP IF IHC ELISA, (https://www.scbt.com/p/ppargamma-antibody-e-8?requestFrom=search); In this paper for IP.
Rabbit anti-FASN, Human, WB IHC IF, (https://www.instrument.com.cn/netshow/SH104673/Q4908007.htm); In this paper for WB.
Rabbit anti-ACC, Human and Mouse, WB IHC IF IP, (https://www.cellsignal.co.uk/products/primary-antibodies/acetyl-coa-carboxylase-c83b10-rabbit-mab/3676?site-search-type=Products&N=4294956287&Ntt=acc&fromPage=plp); In this paper for WB and IHC.
Rabbit anti-ACLY, Human, WB, (https://www.cellsignal.co.uk/products/primary-antibodies/atp-citrate-lyase-antibody/4332?site-search-type=Products&N=4294956287&Ntt=acly&fromPage=plp); In this paper for WB and IHC.
Rabbit anti-GAPDH, Human and Mouse, WB IHC, (https://www.cellsignal.co.uk/products/primary-antibodies/gapdh-d16h11-xp-rabbit-mab/5174?_=1607575634519&Ntt=5174&tahead=true); In this paper for WB.
Rabbit anti-PPARα, Human and Mouse, WB IHC IP, (https://www.ptgcn.com/products/PPARA-Antibody-15540-1-AP.htm); In this paper for WB.
Rabbit anti-PPARδ, Human and Mouse, WB IHC IF, (https://www.ptgcn.com/products/PPARD-Antibody-10156-2-AP.htm); In this paper for WB.
Rabbit anti-SREBF1,Human and Mouse, WB IP IHC IF, (https://www.ptgcn.com/products/SREBF1-Antibody-14088-1-AP.htm); In this paper for WB.
Mouse anti-FLAG Tag, Human and Mouse, WB RIP IP IHC IF FC CoIP, (https://www.ptgcn.com/products/Flag-Tag-Antibody-20543-1-AP.htm); In this paper for WB and IP.
Mouse anti-MYC Tag, Human and Mouse, WB RIP IP IHC IF FC CoIP, (https://www.ptgcn.com/products/MYC-Antibody-60003-2-Ig.htm); In this paper for WB and IP.
Mouse anti-HA Tag, Human and Mouse, WB IP ,(https://www.scbt.com/p/ha-probe-antibody-f-7?requestFrom=search); In this paper for WB.
Mouse anti-His Tag, WB IP, (https://www.scbt.com/p/his-probe-antibody-h-3?requestFrom=search Santa Cruz Biotechnology Cat#SC-8036); In this paper for WB.
Rabbit anti-V5 Tag, WB IP ELISA, (https://www.ptgcn.com/products/V5-tag-Antibody-14440-l-AP.htm); In this paper for WB.
Rabbit anti-USP1, Human, WB IP IHC IF, (https://www.ptgcn.com/products/USP1-Antibody-14346-1-AP.htm); In this paper for WB.

Rabbit anti-USP3, Human and Mouse, WB IP IF ELISA, (https://www.ptgcn.com/products/USP3-Antibody-12490-1-AP.htm); In this paper for WB.
Rabbit anti-USP11, Human and Mouse, WB IP IF ELISA, (https://www.ptgcn.com/products/USP11-Antibody-10244-1-AP.htm); In this paper for WB.
Rabbit anti-USP13,Human and Mouse, WB IP IF ELISA, (https://www.ptgcn.com/products/USP13-Antibody-16840-1-AP.htm); In this paper for WB.
Rabbit anti-USP14, Human and Mouse, WB IP IF ELISA, (https://www.ptgcn.com/products/USP14-Antibody-14517-1-AP.htm); In this paper for WB.
Rabbit anti-USP15,Human and Mouse, WB IP IF ELISA, (https://www.ptgcn.com/products/USP15-Antibody-14354-1-AP.htm); In this paper for WB.
Rabbit anti-USP17, Human, WB, (https://www.abcam.cn/usp17l24-antibody-c-terminal-ab174914.html); In this paper for WB.
Rabbit anti-USP21, Human and Mouse, WB IHC IF ELISA, (https://www.ptgcn.com/products/USP21-Antibody-17856-1-AP.htm); In this paper for WB.
Rabbit anti-USP22,Human and Mouse, WB IHC IF IP, (https://www.sigmaaldrich.com/catalog/product/sigma/hpa044980?lang=zh&region=CN); Rabbit anti-USP24, Human and Mouse, WB IHC IF ELISA, (https://www.ptgcn.com/products/USP24-Antibody-13126-1-AP.htm); In this paper for IHC and IF.
Mouse anti-USP22, WB IP IF ELISA,(https://www.scbt.com/p/usp22-antibody-c-3?requestFrom=search); In this paper for WB and IP.
Rabbit anti-USP24, WB IHC IF ELISA, (https://www.ptgcn.com/products/USP24-Antibody-13126-1-AP.htm); In this paper for WB.
Rabbit anti-USP26, Human and Mouse, WB, (https://www.elabscience.com/p-usp26_polyclonal_antibody-49898.html); In this paper for WB.
Rabbit anti-USP27, Human and Mouse, WB,(https://www.instrument.com.cn/netshow/SH104673/Q4894072.htm); In this paper for WB.
Mouse anti-USP29, Human, WB IP IHC, (https://www.scbt.com/p/usp29-antibody-1a8?requestFrom=search); In this paper for WB.
Rabbit anti-USP33, Human and Mouse, WB IHC IF ELISA, (https://www.ptgcn.com/products/USP33-Antibody-20445-1-AP.htm); In this paper for WB.
Rabbit anti-USP34, Human, WB IHC, (https://www.ptgcn.com/products/USP34-Antibody-18827-1-AP.htm); In this paper for WB.
Rabbit anti-USP36, Human and Mouse, WB IHC IF ELISA, (https://www.ptgcn.com/products/USP36-Antibody-14783-1-AP.htm); In this paper for WB.
Rabbit anti-USP37, Human and Mouse, WB IHC IF ELISA, (https://www.ptgcn.com/products/USP37-Antibody-18465-1-AP.htm); In this paper for WB.
Rabbit anti-USP39, Human and Mouse, WB IHC IF ELISA, (https://www.ptgcn.com/products/USP39-Antibody-23865-1-AP.htm); In this paper for WB.
Rabbit anti-USP46, Human and Mouse, WB IHC IP ELISA,(https://www.ptgcn.com/products/USP46-Antibody-13502-1-AP.htm); In this paper for WB.
Rabbit anti-USP48, Human and Mouse, WB IHC IP, (https://www.ptgcn.com/products/USP48-Antibody-12076-1-AP.htm); In this paper for WB.
Rabbit anti-USP51, Human, WB, (http://www.abnova.com/products/products_detail.asp?catalog_id=H00158880-A01); In this paper for WB.
Rabbit anti-USP54, Human, WB IHC, (https://www.abcam.cn/usp54-antibody-ab151001.html); In this paper for WB.
Rabbit anti-SCD, Human, WB IP IHC, (https://www.abcam.cn/scd1-antibody-epr21963-ab236868.html); In this paper for WB.
Rabbit anti-p-AKT(Ser473), WB IP IHC IF F,(https://www.cellsignal.cn/products/primary-antibodies/phospho-akt-ser473-d9e-xprabbit-mab/4060?site-search-type=Products&N=4294956287&Ntt=4060s&fromPage=plp&_requestid=26905); In this paper for WB.
Rabbit anti-CPT1A, FC IF IHC IP WB ELISA,(https://www.ptgcn.com/products/CPTlA-Antibody-15184-l-AP.htm); In this paper for WB.
Rabbit anti-CPT2, IF IHC IP WB ELISA,(https://www.ptgcn.com/products/CPT2-Antibody-26555-l-AP.htm); In this paper for WB.
Rabbit anti-ACOX1, IHC WB ELISA,(https://www.ptgcn.com/products/AOX-Antibody-10957-l-AP.htm); In this paper for WB.
Rabbit anti-ACADL, IHC, WB,ELISA,(https://www.ptgcn.com/products/ACADL-Specific-Antibody-17526-l-AP.htm); In this paper for WB.
Rabbit anti-ECHS1, IF IHC IP WB ELISA,(https://www.ptgcn.com/products/ECHSl-Antibody-11305-l-AP.htm); In this paper for WB.
Rabbit anti-pVHL, IF WB ELISA, (https://www.ptgcn.com/products/VHL-Antibody-24756-1-AP.htm); In this paper for WB.
Rabbit anti-CUL4B, CoIP IF IHC RIP WB,(https://www.ptgcn.com/products/CUL4B-Antibody-12916-1-AP.htm); In this paper for WB.
Mouse anti-Actin, Human and Mouse, WB IHC IF FC ELISA, (https://www.ptgcn.com/products/ACTB-Antibody-60008-1-Ig.htm); In this paper for WB.

Eukaryotic cell lines

Policy information about cell lines

Cell line source(s)	MHCC-97H, HUH7, Bel-7402, Hep3B, HepG2, SMMC-7721, HEK293T, HCCLM3 and PLC/PRF/5 were purchased from the cell bank of the Committee on Type Culture Collection of the Chinese Academy of Sciences (Shanghai, China). THLE-2 and SNU449 cell lines were purchased from ATCC. MHCC-97L and HB611 were obtained from Liver Cancer Institute, Fudan University (China).
Authentication	MHCC-97H, HUH7, Hep3B, HepG2, HEK-293T, HCC-LM3, ThLE-2, SNU449, PLC/PRF/5 have been authenticated by STR profiling. SMMC-7721,MHCC-97L and HB611 are not authenticated.
Mycoplasma contamination	Cell lines were routinely tested for potential mycoplasma contamination by using commercial mycoplasma detection kits (Lonza, LT07-418). All tests were negative.
Commonly misidentified lines (See ICLAC register)	It was not considered as a misidentified cell line when we started this research. We are not longer use BEL-7402 cell line since it was in the misidentified cell line list.

Animals and other organisms

Policy information about studies involving animals; ARRIVE guidelines recommended for reporting animal research

Laboratory animals	Pathogen-free male athymic nude mice (4-5 weeks old, 18-22 g) were purchased from the Beijing Vital River Laboratory Animal Technology Co., Ltd (Beijing, China). All the mice were housed in a specific pathogen-free (SPF) environment on a 12 h light/dark cycle at temperature 20–25°C and humidity 50–60% at the Institute of Genome Engineered Animal Models for Human Disease of Dalian Medical University.
Wild animals	The study did not involve wild animals.
Field-collected samples	No field-collected samples were used in this study.
Ethics oversight	All animal procedures were conducted in accordance with the guidelines of the Institutional Committee for the Ethics of Animal Care and Treatment in Biomedical Research of Dalian Medical University.

Note that full information on the approval of the study protocol must also be provided in the manuscript.

Human research participants

Policy information about studies involving human research participants

Population characteristics	Informed consent was obtained from all participants in accordance with the Declaration of Helsinki. All samples were collected with the informed consent of the patients and the experiments were approved by research ethics committee at the first affiliated hospital of Dalian Medical University. Human HCC tissue microarrays (Commercialized HCC microarrays) were obtained from Shanghai Outdo Biotech Company or Shanxi ChaoYing Biotechnology Company.<br>The basic characteristics of 10 HCC patients were as follows: 6 female and 4 male, the age range is between 48 and 60, 7 patients were HBV-infected (taking antivirals regularly), no other high-risk recurrence factors, and received no adjuvant therapy (except antiviral therapy) either preoperatively or postoperatively. All patients were diagnosed with HCC by postoperative pathology and were free of other cancers and chronic diseases.<br>The basic characteristics of HLivH180Su11 TMA (samples with death within 3 months after surgery and incomplete clinical information were removed) were as follows: a total of 85 patients, 9 female and 76 male, the age range is between 25 and 73, all with pathological diagnosis of HCC.<br>The basic characteristics of Lv1021 TMA were as follows: a total of 74 patients, 18 female and 56 male, the age range is between 38 and 63, all with pathological diagnosis of HCC.
Recruitment	10 pairs of HCC samples were obtained from the first affiliated hospital of Dalian Medical University (Dalian, China) from June 1st, 2015 to December 1st 2015. All the patients have been diagnosed with primary hepatocellular carcinoma by pathology.<br>We randomly recruited 10 patients with a preoperative clinical diagnosis of HCC, BCLC grade A, and assessed as clinically resectable between June 1 and December 1, 2015. All patients signed an informed consent form for biospecimen use. Due to the small sample size, we did not perform data analysis including survival and clinical characteristics. Only metabolomics and protein expression assays were performed on cancer and paracancerous tissues, and subsequent differential analysis was performed. Therefore, the sample set was somewhat specific, but we further confirmed our findings with subsequent tissue microarrays of large samples.
Ethics oversight	All samples were collected with the informed consent of the patients and the experiments were approved by research ethics committee at the first affiliated hospital of Dalian Medical University.

Note that full information on the approval of the study protocol must also be provided in the manuscript.
